# CUL4B protects kidneys from acute injury by restraining p53/PAI-1 signaling

**DOI:** 10.1038/s41419-024-07299-w

**Published:** 2024-12-18

**Authors:** Kaixuan Liu, Xiaoyu Hao, Yangfan Gao, Zhiyuan Cao, Min Hou, Lining Qin, Yu Song, Molin Wang, Baichun Jiang, Qiao Liu, Yongxin Zou, Yaoqin Gong, Guangyi Liu, Gongping Sun

**Affiliations:** 1 Key Laboratory of Experimental Teratology, Ministry of Education, Department of Histology and Embryology, School of Basic Medical Sciences, Cheeloo College of Medicine, Shandong University, Jinan, Shandong China; 2 Department of Nephrology, Qilu Hospital, Cheeloo College of Medicine, Shandong University, Jinan, Shandong China; 3 Key Laboratory of Experimental Teratology, Ministry of Education, Institute of Molecular Medicine and Genetics, School of Basic Medical Sciences, Cheeloo College of Medicine, Shandong University, Jinan, Shandong China

**Keywords:** Cell death, Acute kidney injury

## Abstract

Acute kidney injury (AKI) caused by nephrotoxins, ischemia reperfusion (IR) or sepsis is associated with high morbidity and mortality. Unveiling new mechanisms underlying AKI can help develop new therapeutic strategy. Cullin 4B (CUL4B) is a scaffold protein in the CUL4B-RING E3 ubiquitin ligase (CRL4B) complex. Here, we demonstrate that CUL4B can protect kidneys from acute injury induced by cisplatin and IR. CUL4B is upregulated in mouse tubular epithelial cells (TECs) after cisplatin treatment or IR. Loss of CUL4B in kidneys exacerbates renal injury, inflammation, and apoptosis of TECs caused by cisplatin and IR. Transcriptome analysis reveals that *Cul4b* deficiency enhances injury-induced PAI-1 expression. CUL4B suppresses PAI-1 expression by promoting polyubiquitination and degradation of p53. Inhibition of either PAI-1 or p53 can prevent the aggravated renal injury and inflammation caused by loss of CUL4B. Our work has identified the kidney-protective role of CUL4B against acute injury.

## Introduction

Acute kidney injury (AKI), defined by an abrupt decrease in kidney function occurring over 7 days or fewer, affects about 20% of hospitalized patients [1]. The common causes of AKI include exposure to nephrotoxins, sepsis, major surgery, renal hypoperfusion, etc. Patients with AKI usually exhibit increased serum creatinine, reduced urinary output, death of tubular epithelial cells (TECs) and inflammation [2]. Currently, therapies for AKI are very limited, making high mortality and poor prognosis of AKI patients. Identifying new regulatory molecules and therapeutic targets for AKI is in urgent need.

The Cullin-RING E3 ubiquitin ligase (CRL) complexes constitute the largest superfamily of E3 ligases [3]. Besides mediating polyubiquitination of protein substrates to direct their degradation, the CRL4B complexes, which use CUL4B as the scaffold protein, can also repress gene transcription by catalyzing monoubiquitination of H2AK119 [4, 5]. CUL4B has been shown to regulate a variety of developmental processes such as embryogenesis, spermatogenesis, osteogenesis, adipogenesis, neurogenesis via assembling the CRL4B complexes [6–12]. In addition, CUL4B plays important roles in diseases. Mutation in *CUL4B* gene is one of the top causes of human X-linked intellectual disability [13, 14]. In many types of cancer, CUL4B functions as an oncogene to promote cancer growth, metastasis and drug resistance [15]. Myeloid depletion of CUL4B exacerbates lipopolysaccharide (LPS)-induced peritonitis and septic shock [16, 17]. Recently, our group has reported that loss of CUL4B in macrophages ameliorates renal injury and fibrosis in diabetic mice by reducing macrophage infiltration [18]. However, whether CUL4B in kidney parenchymal cells participates in regulation of acute or chronic kidney injury is not clear.

Here, we show that CUL4B is upregulated in TECs in kidneys from both mice and patients with AKI. By generating mice with *Cul4b* deleted in kidneys, we demonstrate that CUL4B exerts a kidney-protective effect in response to cisplatin or IR through suppressing the p53-dependent PAI-1 expression.

## Results

### CUL4B is upregulated in kidneys with acute injury

**Fig. 1:**
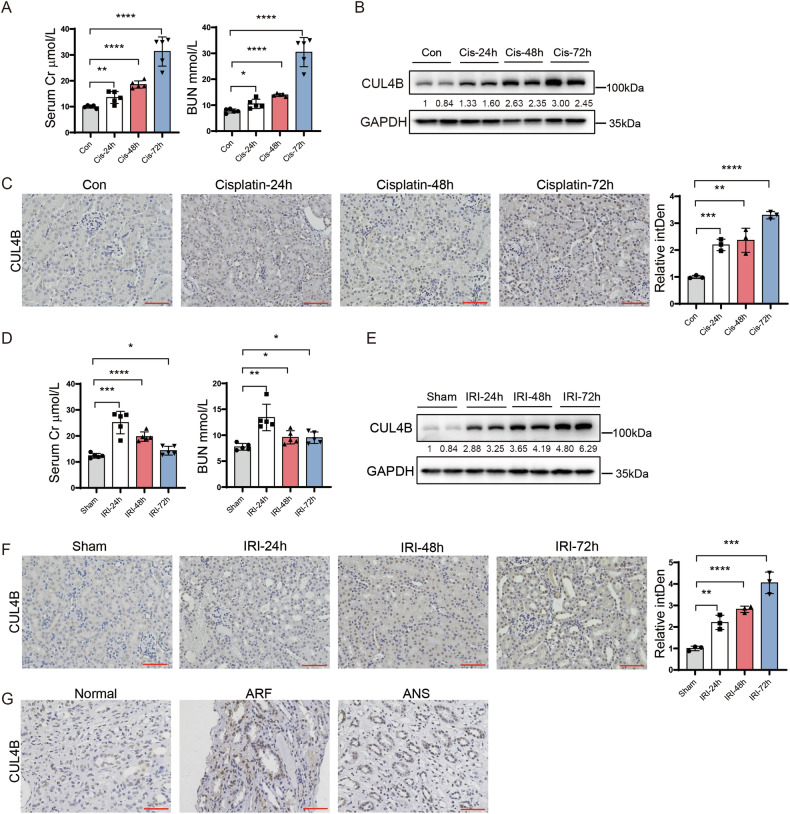
CUL4B is upregulated in kidneys with acute injury. **A** The creatinine (Cr) and BUN levels in the serum collected from mice at 24, 48 or 72 h after peritoneal injection of 20 mg/kg cisplatin (Cis) or at 24 h after injection of the vehicle control (Con). *N* = 5. **B** Western blots showing the level of CUL4B protein in kidneys of the indicated mice. **C** Immunohistochemistry staining of CUL4B in kidneys of the indicated mice. The bar graph shows integrated density (intDen) relative to Con. 5 fields were imaged for each mouse and 3 mice were included in each group. Scale bar, 60 μm. **D** The creatinine (Cr) and BUN levels in the serum collected from mice at 24, 48 or 72 h after IRI or at 24 h after sham operation. *N* = 5. **E** Western blots showing the level of CUL4B protein in kidneys of the indicated mice. **F** Immunohistochemistry staining of CUL4B in kidneys of the indicated mice. The bar graph shows integrated density (intDen) relative to Sham. 5 fields were imaged for each mouse and 3 mice were included in each group. Scale bar, 60 μm. **G** Immunohistochemistry staining of CUL4B in kidneys from patients with acute renal failure (ARF) or acute nephritic syndrome (ANS) or the paracancerous tissues (Normal) obtained from individuals with kidney cancer and without other renal diseases who underwent nephrectomies. Scale bar, 60 μm. Data are presented as mean ± SD. *P < 0.05; **P < 0.01; ***P < 0.001; ****P < 0.0001.

CUL4B is expressed in both glomeruli and renal tubules (Supplementary Fig. S1). To investigate the role of CUL4B in AKI, we first examined the expression of CUL4B in the kidneys of mice after peritoneal injection of cisplatin at a dose of 20 mg/kg. The kidney injury was verified by the elevated levels of blood urea nitrogen (BUN) and creatinine in serum (Fig. 1A) and the increased expression of markers for tubular injury, KIM-1 and NGAL, as well as the tubular damage scoring (Supplementary Fig. S2A–C). We detected time-dependent upregulation of CUL4B in kidneys after cisplatin injection (Fig. 1B). Immunohistochemistry staining revealed that CUL4B was mainly elevated in the TECs (Fig. 1C). To figure out whether CUL4B upregulation is specific to cisplatin-induced injury, we adopted another commonly-used AKI model, ischemia reperfusion injury (IRI) (Fig. 1D, Supplementary Fig. S2D–F). CUL4B was gradually increased in kidneys from 24 to 72 h after IRI (Fig. 1E, F). Moreover, the level of CUL4B in the renal tubules from patients with acute tubular necrosis was much higher than that in the control samples (Fig. 1G). These data together suggest the involvement of CUL4B in AKI.

### *Cul4b* deficiency exacerbates AKI induced by cisplatin or ischemia reperfusion

**Fig. 2:**
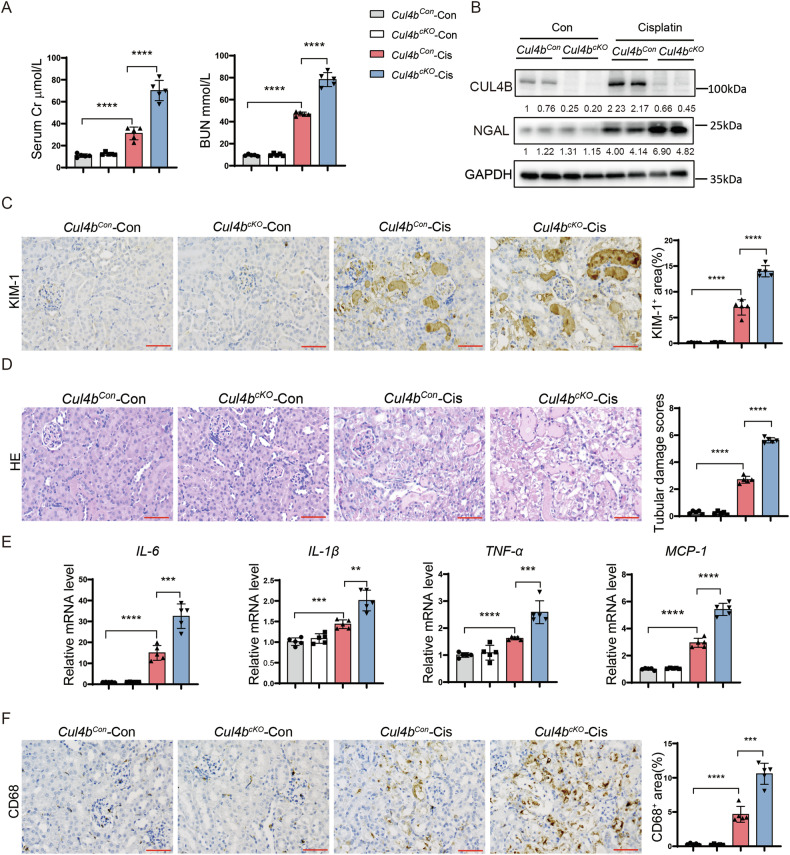
*Cul4b* deficiency exacerbates cisplatin-induced AKI. **A** The creatinine (Cr) and BUN levels in the serum collected from the *Cul4b*^*Con*^ or *Cul4b*^*cKO*^ mice at 72 h after injection of cisplatin (Cis) or the vehicle control (Con). *N* = 5. **B** Western blots of CUL4B and NGAL in the kidneys from the indicated mice. **C** The representative images of immunohistochemistry staining of KIM-1 and quantification of the percentage of KIM-1^+^ area in the indicated kidneys. 5 fields were imaged for each mouse and 5 mice were included in each group. Scale bar, 60 μm. **D** The representative images of Hematoxylin & Eosin (H & E) staining and the tubular damage scores of the kidneys from the indicated mice. 10 fields were imaged for each mouse and 5 mice were included in each group. Scale bar, 60 μm. **E** The mRNA levels of the indicated inflammatory factors in the kidneys. *N* = 5. **F** The representative images of immunohistochemistry staining of the macrophage marker CD68 and quantification of the percentage of CD68^+^ area in kidneys. 5 fields were imaged for each mouse and 5 mice were included in each group. Scale bar, 60 μm. In all bar graphs, the grey bars represent *Cul4b*^*Con*^ mice injected with vehicle control (*Cul4b*^*Con*^-Con); the white bars represent *Cul4b*^*cKO*^ mice injected with vehicle control (*Cul4b*^*cKO*^-Con); the red bars represent *Cul4b*^*Con*^ mice injected with cisplatin (*Cul4b*^*Con*^-Cis); the blue bars represent *Cul4b*^*cKO*^ mice injected with cisplatin (*Cul4b*^*cKO*^-Cis). Data are presented as mean ± SD. *P < 0.05; **P < 0.01; ***P < 0.001; ****P < 0.0001.

To determine the function of CUL4B in AKI, we knocked out *Cul4b* in kidneys by crossing *Pax8-Cre*^*+/–*^ mice with *Cul4b*^*flox/flox*^ mice. Immunohistochemistry staining confirmed depletion of CUL4B in kidneys of *Pax8-Cre*^*+/–*^ *Cul4b*^*flox/Y*^ (*Cul4b*^*cKO*^) mice (Supplementary Fig. S3A). The histological morphology of *Cul4b*^*cKO*^ kidneys was normal (Supplementary Fig. S3B). The body weight, kidney weight and serum creatinine and BUN of the eight-week *Cul4b*^*cKO*^ mice were comparable to those of the age-matched controls (*Cul4b*^*flox/Y*^ mice, designated as *Cul4b*^*Con*^) (Supplementary Fig. S3C). Seventy-two hours after cisplatin injection, *Cul4b*^*cKO*^ mice exhibited significantly higher levels of serum creatinine and BUN than *Cul4b*^*Con*^ mice (Fig. 2A). Loss of CUL4B enhanced cisplatin-induced tubular injury as demonstrated by the elevated expression of NGAL and KIM-1 (Fig. 2B, C) and the increased injury scores (Fig. 2D). Inflammation is an important step in AKI [19]. We found that the *Cul4b*^*cKO*^ kidneys displayed upregulated expression of pro-inflammatory cytokines and chemokines (Fig. 2E) and elevated macrophage infiltration compared to the *Cul4b*^*cKO*^ kidneys (Fig. 2F). The exacerbated renal injury and inflammation caused by *Cul4b* depletion was also observed in mice with IRI (Supplementary Fig. S4). These results indicate that CUL4B protects mice against AKI.

### Loss of CUL4B renders TECs more susceptible to apoptosis

**Fig. 3:**
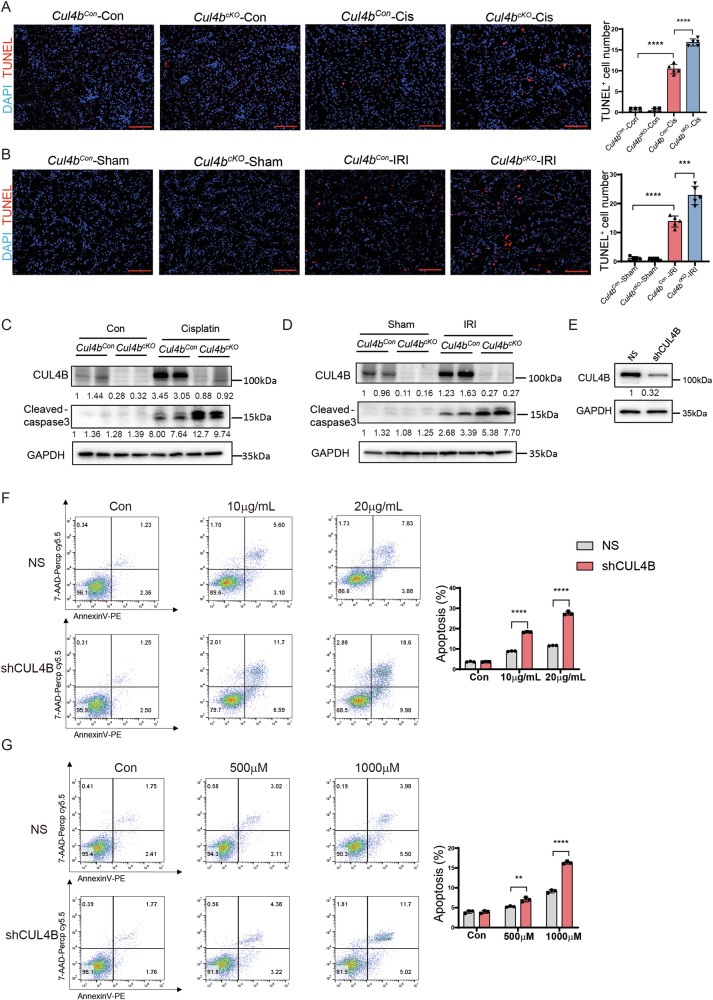
Loss of CUL4B renders TECs more susceptible to apoptosis. **A**, **B** The representative images of TUNEL staining on kidneys and the quantification of the number of TUNEL^+^ cells in each field. 5 fields were imaged for each mouse and 5 mice were included in each group. Scale bar, 85 μm. **C**, **D** Western blots showing the levels of cleaved caspase-3 in kidneys from the indicated groups. **E** Western blots showing the levels of CUL4B protein in HK2 cells expressing control shRNA (NS) or shRNA against *CUL4B* (shCUL4B). The representative images of Annexin V/7-AAD analysis of HK2 cells with or without *CUL4B* knockdown exposed to different doses of cisplatin (**F**) or cobalt chloride (**G**) and the quantification of the percentage of Annexin V^+^ cells. *N* = 3. Data are presented as mean ± SD. *P < 0.05; **P < 0.01; ***P < 0.001; ****P < 0.0001.

Next, we assessed the effect of *Cul4b* knockout on apoptosis in kidneys upon AKI. Without injury, both the *Cul4b*^*cKO*^ and the *Cul4b*^*Con*^ kidneys showed little apoptosis. After cisplatin treatment or IRI, the *Cul4b*^*cKO*^ kidneys exhibited elevated TUNEL^+^ staining (Fig. 3A, B) and increased cleaved caspase-3 compared to the *Cul4b*^*Con*^ kidneys (Fig. 3C, D). We further confirmed the role of CUL4B in apoptosis of TECs by knocking down *CUL4B* in the human proximal tubule epithelial cell line HK2 (Fig. 3E). RNA interference of *CUL4B* resulted in increased apoptosis in response to treatment with cisplatin or cobalt chloride (Fig. 3F, G). These data together suggest that CUL4B protects TECs from apoptosis.

### *Cul4b* deficiency enhances AKI through upregulating PAI-1 expression

**Fig. 4:**
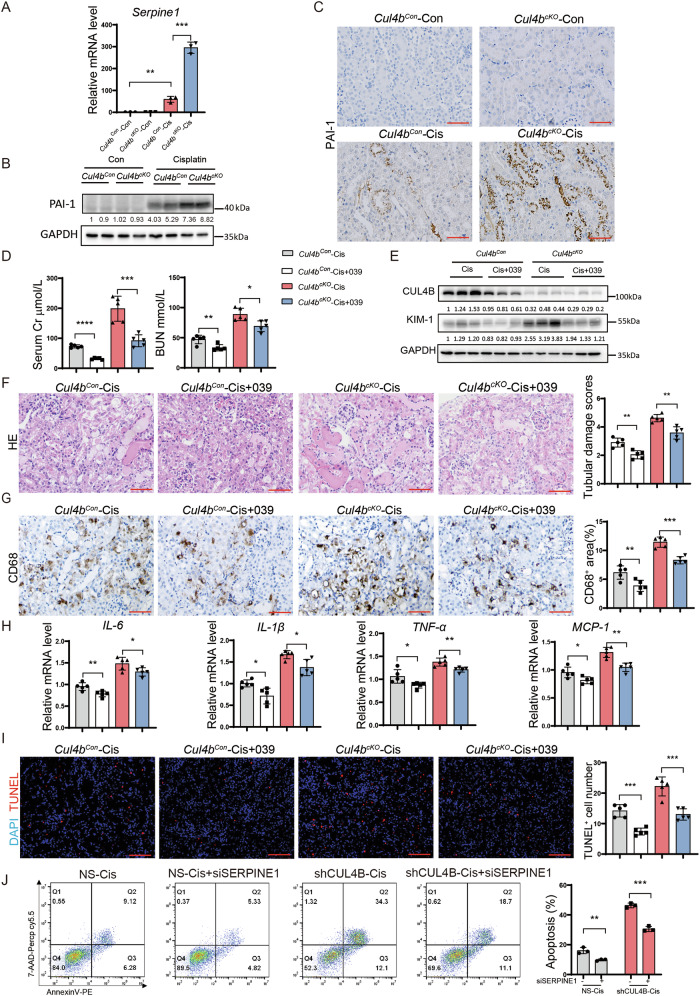
*Cul4b* deficiency enhances AKI through upregulating PAI-1 expression. The levels of *Serpine1* mRNA (**A**) and PAI-1 protein (**B**) in kidneys from the indicated groups. *N* = 3 in (**A**). **C** Immunohistochemistry staining of PAI-1 in kidneys from the indicated groups. Scale bar, 60 μm. **D** The creatinine (Cr) and BUN levels in the serum collected from the *Cul4b*^*Con*^ or *Cul4b*^*cKO*^ mice at 72 h after injection of cisplatin (Cis) with or without pretreatment of PAI-039 (039), an inhibitor for PAI-1. *N* = 5. **E** Western blots showing the levels of CUL4B and KIM-1 in the indicated kidneys. **F** The representative images of H & E staining and the tubular damage scores of the kidneys from the indicated mice. 10 fields were imaged for each mouse and 5 mice were included in each group. Scale bar, 60 μm. **G** The representative images of immunohistochemistry staining of the macrophage marker CD68 and quantification of the percentage of CD68^+^ area in kidneys. 5 fields were imaged for each mouse and 5 mice were included in each group. Scale bar, 60 μm. **H** The mRNA levels of the indicated inflammatory factors in the kidneys. *N* = 5. **I** The representative images of TUNEL staining on kidneys and the quantification of the number of TUNEL^+^ cells in each field. 5 fields were imaged for each mouse and 5 mice were included in each group. Scale bar, 85 μm. In all bar graphs in (**D**)–(**I**), the bars from left to right represent *Cul4b*^*Con*^ mice injected with cisplatin (*Cul4b*^*Con*^-Cis), *Cul4b*^*Con*^ mice pretreated with PAI-039 before injection with cisplatin (*Cul4b*^*Con*^-Cis+039), *Cul4b*^*cKO*^ mice injected with cisplatin (*Cul4b*^*cKO*^-Cis) and *Cul4b*^*cKO*^ mice pretreated with PAI-039 before injection with cisplatin (*Cul4b*^*Con*^-Cis+039), respectively. **J** The representative images of Annexin V/7-AAD analysis of the cisplatin-treated HK2 cells expressing control shRNA (NS) or shRNA against *CUL4B* (shCUL4B) together with or without siRNA against *SERPINE1* (siSERPINE1) and the quantification of the percentage of Annexin V^+^ cells. *N* = 3. Data are presented as mean ± SD. *P < 0.05; **P < 0.01; ***P < 0.001; ****P < 0.0001.

To investigate the mechanism underlying CUL4B regulation of AKI, we performed RNA sequencing on kidneys from the *Cul4b*^*Con*^ and *Cul4b*^*cKO*^ mice with or without cisplatin treatment. Cisplatin treatment resulted in dramatic transcriptome changes in both *Cul4b*^*Con*^ and *Cul4b*^*cKO*^ mice (Supplementary Fig. S5A, B). Compared to the kidneys from the cisplatin-treated *Cul4b*^*Con*^ mice, 561 genes were differentially expressed in the *Cul4b*^*cKO*^ kidneys exposed to cisplatin (Supplementary Fig. S5B). Consistent with the elevated apoptosis and inflammation, genes upregulated in the cisplatin-treated *Cul4b*^*cKO*^ group were enriched in GO terms related to apoptosis and inflammation (Supplementary Fig. S5C). Among the differentially expressed genes, *Serpine1*, which encodes plasminogen activator inhibitor-1 (PAI-1), drew our attention. PAI-1 is a member of the serine protease inhibitor family that can suppress urokinase-type plasminogen activator to inhibit fibrinolysis. PAI-1 is rarely expressed in healthy kidneys but has been reported upregulated in kidneys with acute or chronic injury [20]. RNA sequencing revealed strong upregulation of *Serpine1* mRNA in kidneys from the cisplatin-treated *Cul4b*^*cKO*^ mice compared to that in the cisplatin-treated *Cul4b*^*Con*^ group, which was confirmed by RT-qPCR (Fig. 4A). Western blotting and immunohistochemistry staining demonstrated that PAI-1 protein was barely presented in the uninjured kidneys but induced in the TECs by cisplatin treatment. Loss of CUL4B dramatically elevated the injury-induced PAI-1 in kidneys (Fig. 4B, C). The enhanced PAI-1 expression by *Cul4b* deficiency was also observed in kidneys with IRI (Supplementary Fig. S6). To determine whether loss of CUL4B exacerbates AKI through upregulating PAI-1 expression, we evaluated the effect of the PAI-1 inhibitor tiplaxtinin (PAI-039) on the cisplatin-induced AKI in the *Cul4b*^*Con*^ and the *Cul4b*^*cKO*^ mice. Treatment with PAI-039 significantly ameliorated renal injury in the cisplatin-treated *Cul4b*^*cKO*^ mice as demonstrated by the reduced BUN and creatinine levels in serum (Fig. 4D), the downregulated KIM1 protein (Fig. 4E) and decreased pathological injury scores (Fig. 4F). PAI-039 also reduced macrophage infiltration and expression of inflammatory factors in the cisplatin-treated *Cul4b*^*cKO*^ group (Fig. 4G, H). We further evaluated the effect of PAI-1 inhibition on apoptosis. Inhibition of PAI-1 decreased the number of TUNEL^+^ cells in the kidneys from the cisplatin-treated *Cul4b*^*cKO*^ mice (Fig. 4I). In addition, knocking down *SERPINE1* markedly reduced the cisplatin-induced apoptosis in *CUL4B* knockdown cells (Fig. 4J). Taken together, these data suggest that CUL4B protects kidneys from cisplatin-induced renal injury, inflammation and apoptosis through suppressing PAI-1 expression.

### CUL4B suppresses PAI-1 expression by promoting p53 degradation

**Fig. 5:**
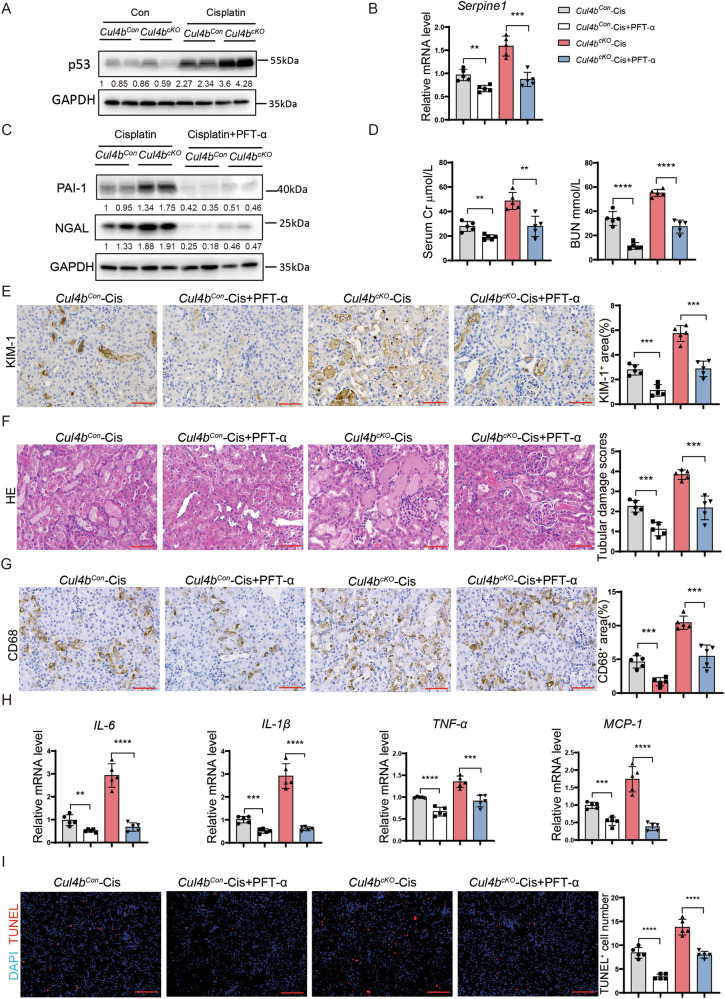
CUL4B suppresses PAI-1 expression by upregulating p53. **A** Western blots showing the level of p53 in the indicated kidneys. **B** The level of *Serpine1* mRNA in kidneys from the cisplatin (Cis)-treated *Cul4b*^*Con*^ or *Cul4b*^*cKO*^ mice with or without pretreatment of PFT-α, an inhibitor for p53. *N* = 5. **C** Western blots showing the levels of PAI-1 and NGAL in the indicated kidneys. **D** The creatinine (Cr) and BUN levels in the serum collected from the indicated mice. *N* = 5. **E** The representative images of immunohistochemistry staining of KIM-1 in kidneys and the quantification of the percentage of KIM-1^+^ area. 5 fields were imaged for each mouse and 5 mice were included in each group. Scale bar, 60 μm. **F** The representative images of H & E staining and the tubular damage scores of the kidneys from the indicated mice. 10 fields were imaged for each mouse and 5 mice were included in each group. Scale bar, 60 μm. **G** The representative images of immunohistochemistry staining of the macrophage marker CD68 and quantification of the percentage of CD68^+^ area in kidneys. 5 fields were imaged for each mouse and 5 mice were included in each group. Scale bar, 60 μm. **H** The mRNA levels of the indicated inflammatory factors in the kidneys. *N* = 5. **I** The representative images of TUNEL staining on kidneys and the quantification of the number of TUNEL^+^ cells in each field. 5 fields were imaged for each mouse and 5 mice were included in each group. Scale bar, 85 μm. In all bar graphs, the bars from left to right represent *Cul4b*^*Con*^ mice injected with cisplatin (*Cul4b*^*Con*^-Cis), *Cul4b*^*Con*^ mice pretreated with PFT-α before injection with cisplatin (*Cul4b*^*Con*^-Cis+PFT-α), *Cul4b*^*cKO*^ mice injected with cisplatin (*Cul4b*^*cKO*^-Cis) and *Cul4b*^*cKO*^ mice pretreated with PFT-α before injection with cisplatin (*Cul4b*^*Con*^-Cis+PFT-α), respectively. Data are presented as mean ± SD. *P < 0.05; **P < 0.01; ***P < 0.001; ****P < 0.0001.

The elevated *Serpine1* mRNA in the cisplatin-injured *Cul4b*^*cKO*^ kidneys indicates CUL4B regulates PAI-1 at the transcription level. p53 has been reported as a transcription activator for *Serpine1* [21–23]. P53 is also a substrate for the CRL4B complex in cancer cells and fibroblasts [24–26]. To determine whether p53 mediates CUL4B suppression of PAI-1 in injured kidneys, we first assessed the level of p53 in kidneys after injury caused by cisplatin or IR. As reported in literature [27], p53 protein was upregulated in the injured kidneys. Importantly, the injured *Cul4b*^*cKO*^ kidneys displayed dramatic elevation of p53 protein compared to the injured *Cul4b*^*Con*^ kidneys (Fig. 5A, Supplementary Fig. S7A). Next, we treated the *Cul4b*^*Con*^ and the *Cul4b*^*cKO*^ mice with Pifithrin-α (PFT-α), which inhibits the transcription regulatory activity of p53, before cisplatin treatment or IR. Inhibition of p53 abolished the difference in the levels of *Serpine1* mRNA and PAI-1 protein between the *Cul4b*^*Con*^ and the *Cul4b*^*cKO*^ kidneys (Fig. 5B, C, Supplementary Fig. S7B, C), suggesting that *Cul4b* knockout increased *Serpine1* expression by upregulating p53. Moreover, inhibition of p53 prevented the exacerbated kidney injury (Fig. 5C–F, Supplementary Fig. S7C–F), inflammation (Fig. 5G, H, Supplementary Fig. S7G, H) and apoptosis (Fig. 5I & Supplementary Fig. S7I) in the *Cul4b*^*cKO*^ mice after cisplatin treatment or IRI. To further confirm the role of p53 in CUL4B regulation of PAI-1, we interfered *TP53* expression in HK2 cells with *CUL4B* knockdown. Knocking down *TP53* suppressed both the increased *SERPINE1* mRNA and the elevated apoptosis induced by reduced CUL4B (Supplementary Fig. S8). These results together indicate that CUL4B inhibits PAI-1 expression and ameliorates AKI by restraining p53 level.

**Fig. 6:**
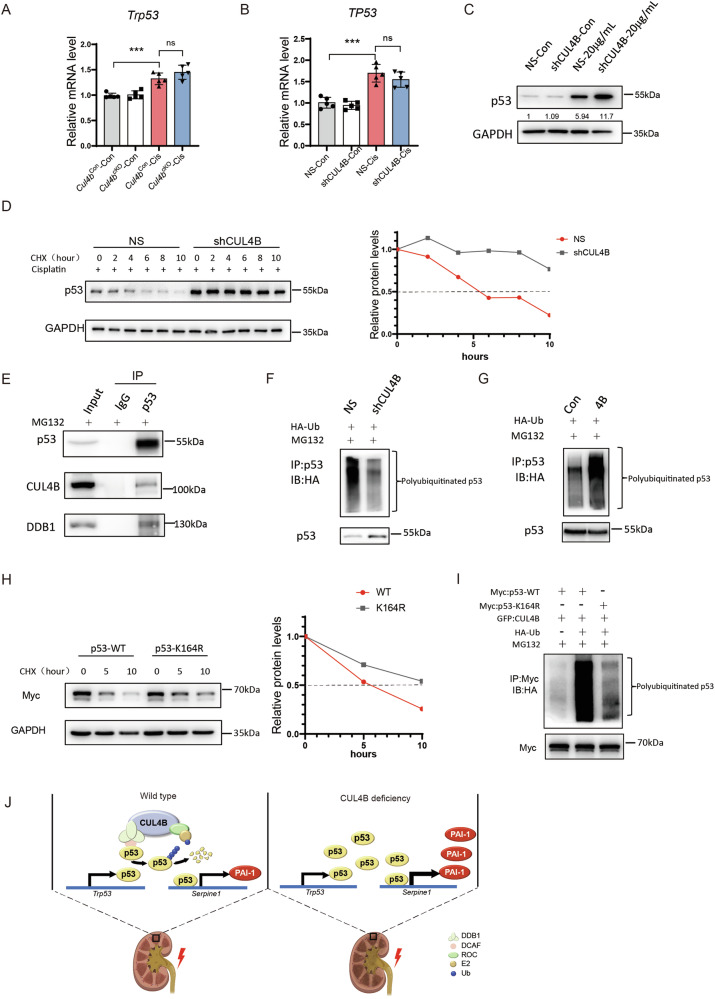
CRL4B complex catalyzes polyubiquitination of p53 at K164 to promote its degradation. **A** The level of *Trp53* mRNA in kidneys from the indicated mice. *N* = 5. **B** The level of *TP53* mRNA in the indicated HK2 cells. *N* = 5. Data are presented as mean ± SD. *P < 0.05; **P < 0.01; ***P < 0.001; ****P < 0.0001. ns: no significance. **C** Western blots showing the level of p53 protein in HK2 cells with *CUL4B* stably knocked down (shCUL4B) or control HK2 cells (NS) treated with 20 μg/ml cisplatin (20 μg/ml) or vehicle control (Con). **D** Western blots showing the level of p53 protein in the cisplatin-treated HK2 cells with or without *CUL4B* knocked down at different time points after treating with CHX and the quantification of the protein levels. **E** The co-immunoprecipitation (IP) assay showing p53 interacts with CUL4B and DDB1 in HK2 cells treated with cisplatin and MG132. Ubiquitination assay showing ubiquitination of p53 in HK2 cells stably knocking down *CUL4B* (**F**) or overexpressing *CUL4B* (**G**). Cells were transfected with HA-Ub plasmid and treated with MG132. The lysates were immunoprecipitated with anti-p53 antibody. **H** Western blots showing the level of wild type or K164R mutant p53 in HEK293T cells at different time points after treating with CHX and the quantification of the protein levels. Cells were transfected with 5×Myc: p53-WT or 5×Myc: p53-K164R. **I** Ubiquitination assay showing the levels of polyubiquitination on wild type or K164R mutant p53 in CUL4B-overexpressing HEK293T cells. Cells were transfected with the indicated plasmids and treated with MG132. The lysates were immunoprecipitated with anti-Myc antibody. **J** Schematic showing the molecular mechanism underlying CUL4B regulation of AKI.

To figure out how CUL4B suppresses p53 in response to kidney injury, we first measured the mRNA level of *Trp53* in the kidneys with or without injury. While cisplatin treatment induced upregulation of *Trp53* mRNA, knockout of *Cul4b* showed no effect (Fig. 6A). In cisplatin-treated HK2 cells, we also observed elevation in p53 protein but not in mRNA after *CUL4B* knockdown (Fig. 6B, C). These data suggest that after kidney injury CUL4B regulates p53 at the post-transcriptional level. To determine whether the CRL4B complex mediates degradation of p53 in kidney cells, we first performed cycloheximide (CHX) chasing experiments to evaluate the effect of *CUL4B* knockdown on p53 degradation. We found that knocking down *CUL4B* in HK2 cells under cisplatin treatment resulted in stabilization of p53 (Fig. 6D). Next, through co-immunoprecipitation, we detected physical interaction between CUL4B, DDB1 and p53 in HK2 cells treated with cisplatin (Fig. 6E). Moreover, knocking down *CUL4B* reduced while overexpression of *CUL4B* increased polyubiquitination of p53 in the cisplatin-treated HK2 cells (Fig. 6F, G). To determine which lysine in p53 is ubiquitinated by CRL4B complex, we purified p53 from cells with CUL4B overexpressed and applied it to mass spectrometry analysis. Lysine 164 (K164) was identified as the major ubiquitination target residue of CRL4B (Supplementary Fig. S9). We then mutated K164 in p53 protein to arginine (R). Compared to wild type p53, the K164R mutant was more stable (Fig. 6H) and exhibited dramatically reduced polyubiquitination in CUL4B-overexpressing cells (Fig. 6I), suggesting that CRL4B regulates p53 stability mainly through ubiquitinating K164 in p53 protein. These data together support that CRL4B complex promotes p53 ubiquitination and degradation in injured kidney cells.

## Discussion

CUL4B has been identified as important regulators in cancer, X-linked intellectual disability and acute inflammatory diseases like peritonitis [13–17]. Yet, the role of CUL4B in kidney diseases is poorly investigated. Previously, our group has reported that loss of CUL4B in myeloid cells reduces infiltration of macrophages into diabetic kidneys, which in turn ameliorates kidney injury and fibrosis [18]. In this study, we, for the first time, demonstrate that CUL4B can protect kidneys from acute injury caused by cisplatin or IR. Upon exposure to cisplatin or IR, CUL4B is elevated in the kidney to promote degradation of p53, which slows down the induced elevation of p53 and the subsequent PAI-1 expression to restrain the injury (Fig. 6J).

In this study, we found that *Cul4b* deficiency upregulated cisplatin or IR-induced PAI-1 expression in TECs. More importantly, the elevated PAI-1 expression accounts for the aggravated tubular injury and renal inflammation caused by loss of CUL4B. Previous reports have linked PAI-1 to AKI. Upregulation of urinary PAI-1 (uPAI-1) was detected in animal models or patients with AKI induced by sepsis, nephrotoxin or IR [28, 29]. In healthy kidneys, PAI-1 is rarely expressed, but elevated PAI-1 expression is common in injured kidneys [30]. Single-cell transcriptome analysis of kidneys from mice injected with LPS revealed that PAI-1 was mainly upregulated in endothelial cells [31]. Consistently, in vitro treatment of LPS only resulted PAI-1 upregulation in endothelial cells but not in TECs, suggesting that endothelial cells are responsible for PAI-1 production in LPS-injured kidneys [29]. However, in kidneys from murine with STZ-induced diabetes or unilateral ureteral obstruction, PAI-1 protein was upregulated in the tubules [32, 33]. Through in situ renal perfusion, Paniagua-Sancho et al. showed that the uPAI-1 in rat models with cisplatin, gentamicin or IR-induced AKI was produced by the damaged tubules and secreted into the urine [28]. In our work, by immunohistochemistry, we also found that in both the cisplatin and IR-injured kidneys PAI-1 was elevated in the TECs. The abovementioned work suggests that although PAI-1 is commonly upregulated in kidneys with acute injury, the cells responsible for producing PAI-1 vary in different AKI models. Exposure to nephrotoxins, IR or high glucose results in upregulation of PAI-1 in TECs, while LPS triggers PAI-1 expression in endothelial cells. In this study, we demonstrated that treatment with PAI-1 inhibitor ameliorated tubular injury and inflammation in both *Cul4b*^*Con*^ and *Cul4b*^*cKO*^ kidneys. Similar pro-injury and pro-inflammation role of PAI-1 has also been reported in models for other kidney diseases. For example, treatment with PAI-1 inhibitor or knockout of *Serpine1* prevented tubular injury, apoptosis and inflammation in kidneys after LPS treatment or IR [29, 34, 35].

PAI-1 has been reported to suppress spontaneous or induced apoptosis in a variety of cell types including pulmonary fibroblasts [36, 37], endothelial cells [38], neutrophils [39], neurons [40] and vascular smooth muscle cells [41]. However, in some other cell types, PAI-1 exerts a pro-apoptotic effect. For example, Jiang et al. showed that PAI-1 was elevated with age in both pulmonary fibroblasts and in alveolar type 2 (ATII) cells. It protected fibroblasts from bleomycin or hydrogen peroxide-induced apoptosis but promoted apoptosis through upregulation of p53 in ATII cells [37]. By applying PAI-1 inhibitor to mice with cisplatin treatment or IRI, we showed that inhibition of PAI-1 suppressed the enhanced tubular apoptosis caused by loss of CUL4B in response to cisplatin treatment or IR. In vitro, knocking down *Serpine1* also reduced cisplatin-induced apoptosis in HK2 cells with or without *CUL4B* knockdown, indicating that PAI-1 has autonomous effect on apoptosis in TECs. Consistent with our finding, Gifford et al. reported that overexpression of PAI-1 in HK2 cells induced apoptosis. They found that the PAI-1-induced apoptosis was mediated by upregulation of p53 [42]. Interestingly, p53 is a known transcription factor that can activate transcription of *Serpine1* gene. In this study, we found that inhibition of p53 abolished upregulation in *Serpine1* mRNA and PAI-1 protein in *Cul4b* knockout kidneys, suggesting that p53 mediates CUL4B regulation of PAI-1. Therefore, p53 and PAI-1 may form a positive feedback loop to aggravate apoptosis in TECs. Disturbing of this loop, as what the injury-induced CUL4B does, can reduce tubular injury.

The CRL4B complex can promote protein degradation by polyubiquitinating protein substrates or repress gene transcription by catalyzing monoubiquitination of H2AK119 [15]. We found that in TECs, the CRL4B complex catalyzed polyubiquitination of p53 and promoted its degradation. And we identified that CRL4B catalyzes polyubiquitination of the K164 residue in p53. CRL4B-mediated p53 degradation also occurs in cancer cells, underlying the pro-tumor effect of CUL4B [24, 26]. CUL4B can protect fibroblasts from radiation-induced senescence by promoting degradation of p53 [25]. These studies, together with our work, indicate that p53 may be a common substrate in different cell types.

In summary, our work, for the first time, reveal that CUL4B protects against AKI associated with cisplatin or IR. The nephroprotective effect of CUL4B is achieved by suppression of p53/PAI-1 signaling, which leads to reduced apoptosis and inflammation. Modulating this pathway may have therapeutic implications to reduce mortality and improve the prognosis after AKI.

## Materials and methods

### Human renal biopsy samples

Renal biopsies were obtained from Department of Pathology, Qilu Hospital of Shandong University. Patients with acute renal failure or acute nephritis syndrome were diagnosed by Department of Nephrology, Qilu Hospital of Shandong University. The control samples were the paracancerous tissues from the patients with kidney cancers who underwent tumor nephrectomies. All the investigation was conducted in accordance with the Declaration of Helsinki and were approved by the Research Ethics Committee of Shandong University. The informed consent was obtained.

### Mouse

All animal experiments were performed in accordance with the protocol approved by the Institutional Animal Care and Use Committee, School of Basic Medical Sciences, Shandong University. C57BL/6J mice were purchased from Beijing Vital River Laboratory Animal Technology Co., Ltd. Floxed *Cul4b* mice [6] were crossed to *Pax8-Cre* mice (The Jackson Laboratory, Strain# 028196) to generate *Pax8-Cre*^*+/-*^*; Cul4b*^*flox/Y*^ (*Cul4b*^*cKO*^) and *Cul4b*^*flox/Y*^ (*Cul4b*^*Con*^) mice. The mice were housed in a specific-pathogen-free facility in plastic cages at 22 °C and 40–50% humidity, with a 12/12 h light-dark cycle. Mice with the same genotype were allocated randomly into different treatment groups.

### AKI mouse model

For cisplatin-induced AKI model, 8 to 10-week-old male mice were injected peritoneally with cisplatin (dissolved in 10% DMSO) at a dose of 20 mg per kg of mouse weight. Mice injected with the same volume of 10% DMSO were served as vehicle control. IRI was performed as previously reported [43]. In brief, after anesthetized with pentobarbital sodium, the mice were placed on a heat pad of 37 °C and a midline abdominal incision was made. The bilateral renal pedicles were clipped for 35 min with vascular clamps (FINE SCIENCE TOOLS, Cat#18055-03). At the end of the ischemia, the clamps were removed to allow reperfusion. Then the abdominal incision was closed, and the mice were placed in a 37 °C incubator to allow recovery. At the desired time after injury, the blood samples were collected from anesthetized mice and serum creatinine and BUN levels were measured by KingMed Diagnostics (Jinan, China). Then the mice were sacrificed, and kidneys were collected, weighted and stored in –80 °C or fixed in 4% paraformaldehyde (PFA) (Servicebio, Cat#G1101).

### Tubular damage scoring

After 24 h fixation in 4% PFA at room temperature, the kidney tissues were embedded in paraffin. The embedded tissues were cut into 4 μm thick sections, adhered onto glass slides, deparaffinized, and rehydrated by decreasing ethanol concentrations. Tissue-embedded slides were stained with hematoxylin and eosin (H & E) and imaged on a microscope (KEYENEC, Cat#BZ-X810). The tubular scoring was performed double blinded according to the presence and the extent of TEC flattening, brush border loss, cell membrane bleb formation, interstitial edema, cytoplasmic vacuolization, necrosis and tubular lumen obstruction [44].

### Immunohistochemistry and immunofluorescent staining

For immunostaining, tissue-embedded slides were washed with PBS, boiled in citrate solution, and blocked with 10% goat serum before overnight incubation at 4 °C with primary antibodies. For immunohistochemistry, after incubation with primary antibodies, the sections were applied with Immunohistochemistry kit (ZSGB-Bio, Cat# PV9001 and PV9000). For immunofluorescent staining, after incubation with primary antibodies, the slides were stained with secondary antibodies (Invitrogen, Cat# A11029 and A11011) for 60 min at room temperature in the dark. Nucleus was labeled with DAPI (Abcam, Cat# ab104139). The TUNEL staining was performed using TUNEL kit (KeyGEN BioTECH, Cat# KGA1405-50) according to the manufacturer’s instruction. Images were collected with a microscope (KEYENEC, Cat#BZ-X810). The analyses of the images were performed using ImageJ. All the antibodies used are listed in Supplementary Table S1.

### Cell culture

HK2 cells were purchased from FuHeng Biology (Cat# FH0228). Cells were maintained in a humidified atmosphere at 37 °C with 5% CO_2_ and routinely tested for Mycoplasma. To generate HK2 cells stably expressing control shRNA or shRNA targeting *CUL4B*, the shRNA sequence [45] was inserted into pGIPZ vector. The constructs were packed into lentivirus and applied to HK2 cells. Forty-eight hours after infection, the cells were subjected to selection with Blasticidin S hydrochloride (Solarbio, Cat# B9300). To generate HK2 cells stably overexpressing CUL4B, lentivirus carrying CUL4B were applied to HK2 cells. The infected cells were selected with Puromycin (InvivoGen, Cat# QLL-44-04). When evaluating effects of different treatments, cells were randomly allocated into different groups.

### Transfection

siRNA transfection was performed using Lipofectamine™ RNAiMAX (Invitrogen, Cat# 137781050). Cells were collected at 24 or 48 h after transfection. Plasmid transfection was performed using Lipofectamine™ 3000 (Invitrogen, Cat# L3000015). Cells were collected at 24 h after transfection.

### Annexin V/7-AAD analysis of apoptosis

Cells were collected and suspended in PBS. The Annexin V-PE/7-AAD staining was performed using Apoptosis Detection Kit (Vazyme, Cat# A213-02) according to the manufacturer’s manual. The stained cell suspension was then applied to flow cytometry analysis using CytoFLEX (Beckman Coulter). The data were analyzed using FlowJo 10.8.1 software.

### CHX chasing experiment

To evaluate protein stability, 50 μg/ml CHX (GLPBIO, Cat# GC17198) were applied to cells to inhibit protein synthesis. Cells were collected and lysed in RIPA buffer (Sigma, Cat# R0278) supplemented with protease inhibitor (Solarbio, Cat# P0100). The protein samples were applied to Western blotting.

### Ubiquitination assay

HK2 or HEK293T Cells were transfected with plasmids and treated with 20 μM MG132 for 5 h at 48 h post transfection. Cells were then lysed and precipitated with anti-Myc antibody or anti-p53 antibody. The precipitates were applied to Western blotting.

### Identification of ubiquitination sites

HEK293T cells were transfected with plasmids expressing 5×Myc-p53, HA-Ub and GFP-CUL4B. Forty-eight hours after transfection, cells were treated with 20 μM MG132 for 5 h. Then cells were collected and lysed. p53 was precipitated with anti-Myc antibody and applied to 10% SDS-PAGE. After Coomassie Blue staining, the protein bands were cut and sent to mass spectrometry analysis. Mass spectrometry analysis was performed by Qinglianbio Biotech (Beijing China). Site-Directed Mutagenesis kit (NEB, Cat# E0552S) was used to generate K164R mutant of p53.

### Co-immunoprecipitation

The cells were lysed in lysis buffer containing 150 mM NaCl, 50 mM Tris-HCl (pH7.4), 1% Triton X-100. After centrifugation, the supernatant was collected and incubated with CUL4B antibody, p53 antibody or control IgG (listed in Supplementary Table S1) at 4 °C overnight. Protein A/G Magnetic beads (Vazyme, Cat# PB101) were added to the protein/antibody mixture and incubated at room temperature for 2 h. The beads were then washed with PBST buffer (PBS containing 0.5% Tween-20) for 5 times and boiled in loading buffer for 10 min. The supernatant was subjected to Western blotting.

### Western blotting

Protein samples from kidney tissues were collected by homogenizing the frozen tissues and lysing in RIPA buffer. Protein samples from cells were prepared as mentioned in the above sections. Protein concentrations were quantified using BCA kit (Vazyme, Cat# E112-02). Equal mass of protein samples and protein markers (Beyotime, Cat# P0069; Vazyme, Cat# MP102) was applied to 10% gels, transferred to PVDF membrane (Millipore, Cat# ISEQ00010), and probed with primary antibodies and secondary antibodies. The bands were detected using Chemiluminescent HRP Substrate (Millipore, Cat# WBKLS0100) and imaged using Tanon 5200 Multi.

### RNA isolation and RT-qPCR

Total RNA was extracted with Trizol reagents (Invitrogen, Cat# 15596018). Reverse transcription was performed with HiScript RT SuperMix for qPCR kit (Vazyme, Cat# R32). RT-qPCR was run using 2×ChamQ SYBR Mix (Vazyme, Cat# Q411) on Analytikjena qTOWER. Primer sequences are listed in Supplementary Table S2.

### RNA sequencing

RNA sequencing was performed using kidney tissues from *Cul4b*^*Con*^ and *Cul4b*^*cKO*^ mice collected at 72 h after treatment with cisplatin or vehicle controls. 3 mice were included in each group. The sequencing was performed by Beijing Biomarker Technologies Co., LTD (Beijing, China) and analyzed using the BMKCloud Bioinformatics analysis platform.

### Statistical analysis

All data are presented as mean ± SD. Statistical analysis was done using GraphPad Prism 8. Statistical significance was determined using unpaired two-tailed *t*-test for two-sample comparison or one-way ANOVA with Tukey’s multiple comparisons test for comparison between three or more groups. The assumption of equal variance was validated by F-test. A *P* value <0.05 was considered significant. The sample sizes were chosen empirically based on the observed effects and previous reports. The sample size for each experiment is listed in the figure legends. When scoring tubular injury and analyzing data of RT-qPCR and immunohistochemistry, the investigators were blinded to the group allocation.

## Supplementary information

Supplementary figures and tables

original images of Western blots

## Data Availability

The raw data of RNAseq were deposited at NCBI Bioproject with accession number PRJNA1108044. The original images of Western blots were provided as a supplementary file. All the other raw data supporting the findings of this study are available from the corresponding authors upon request.
