# Supplementary material for: CUL4B protects kidneys from acute injury by restraining p53/PAI-1 signaling
Source: Cell Death Dis. 2024 Dec 18;15(12):915. doi: 10.1038/s41419-024-07299-w (PMC11655551; doi:10.1038/s41419-024-07299-w)
Supplement: Supplementary file 1 — Supplementary figures and tables [file 41419_2024_7299_MOESM1_ESM.pdf]

CUL4B protects kidneys from acute injury by restraining p53/PAI-1 signaling

Liu et al.

This file contains the following contents.

Supplementary figures S1-S9 and their legends.

Supplementary table S1-S2

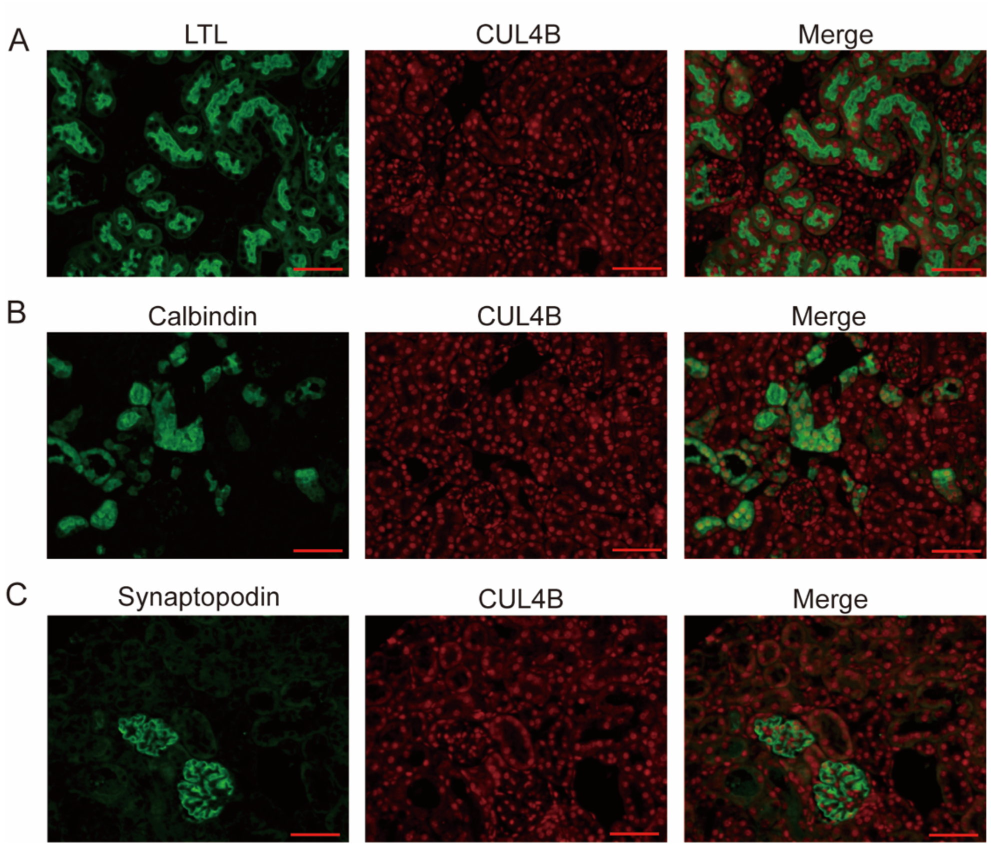

Supplementary figure S1. CUL4B is ubiquitously expressed in glomeruli and renal tubules.

Immunofluorescent staining showing expression of CUL4B in the proximal tubules (marked by LTL), the distal tubules (marked by Calbindin) and podocytes (marked by Synaptopodin). Scale bar, 60 μm.

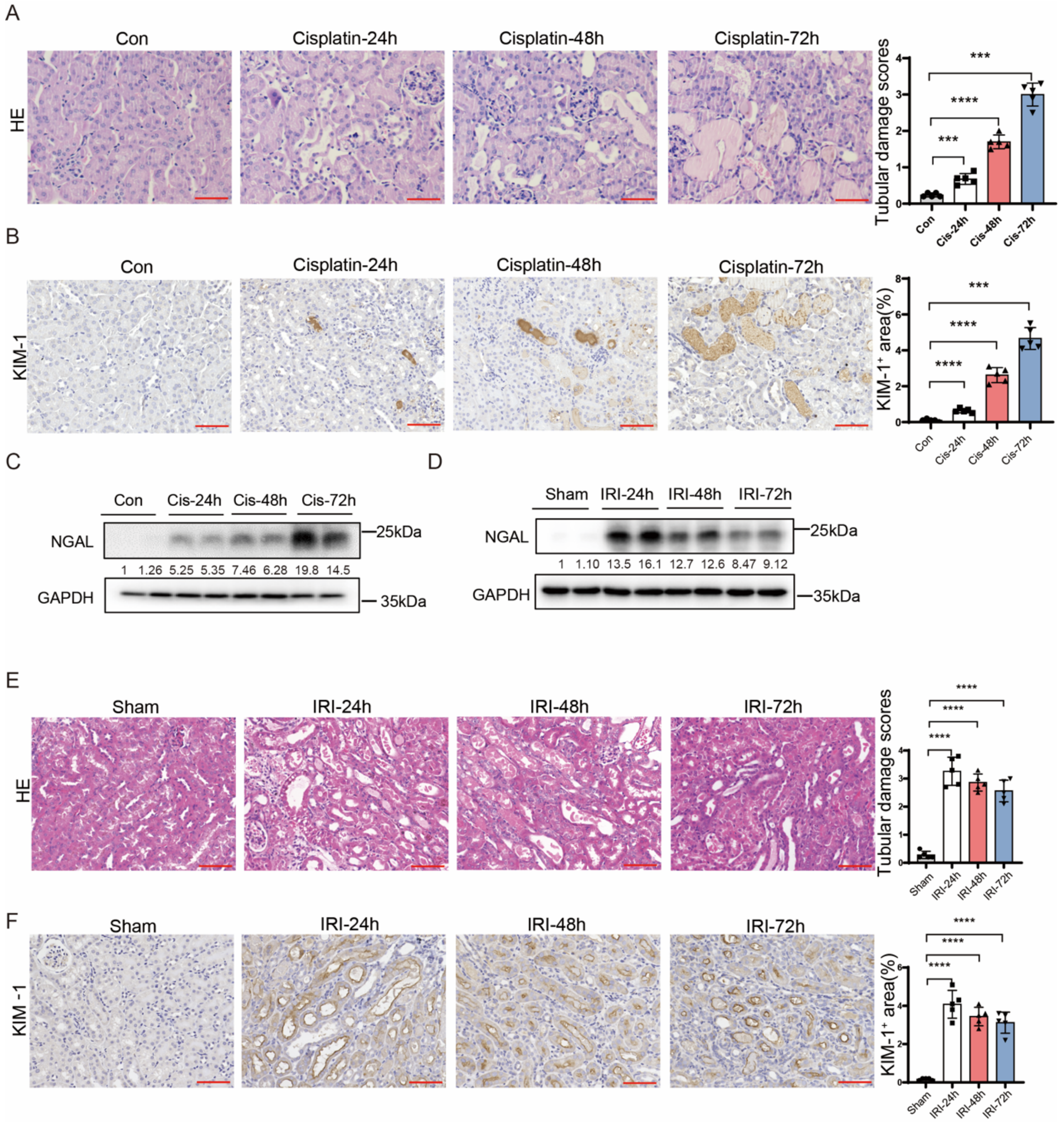

Supplementary figure S2. Cisplatin and IR induce AKI.

A & E) The representative images of H & E staining and the tubular damage scores of the kidneys from the indicated mice. 10 fields were imaged for each mouse and 5 mice were included in each group. Scale bar, 60 μm. B & F) The representative images of immunohistochemistry staining of KIM-1 and quantification of the percentage of KIM-1^{+} area in the indicated kidneys. 5 fields were imaged for each mouse and 5 mice were included in each group. Scale bar, 60 μm. C & D) Western blots showing the level of NGAL in the indicated kidneys. Data are presented as mean ± SD. *: $P< 0.05$; **: $P<$

0.01; ***: *P*< 0.001; ****: *P*< 0.0001.

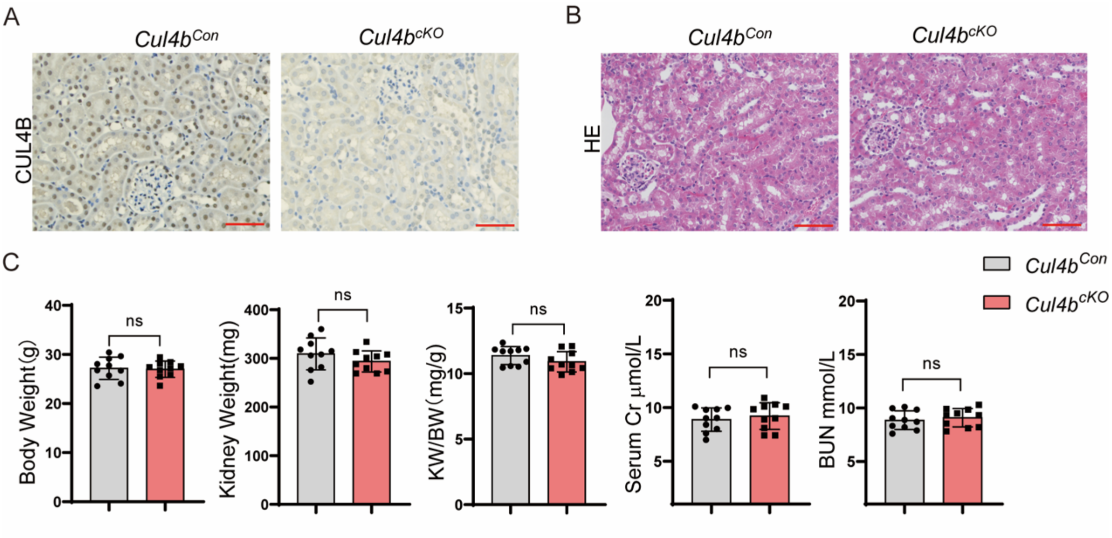

Supplementary figure S3. Knockout of *Cul4b* does not affect renal morphology and function at 8 weeks old.

A) The representative images of immunohistochemistry of CUL4B in *Cul4b*^{Con} and *Cul4b*^{cKO} mice. Scale bar, 60 μm. B) The representative images of H & E staining of *Cul4b*^{Con} and *Cul4b*^{cKO} kidneys. Scale bar, 60 μm. C) The body weight, the kidney weight, the ratio between kidney weight (KW) and body weight (BW), and the level of creatinine (Cr) and BUN in serum of *Cul4b*^{Con} and *Cul4b*^{cKO} mice. N=10. ns: no significance.

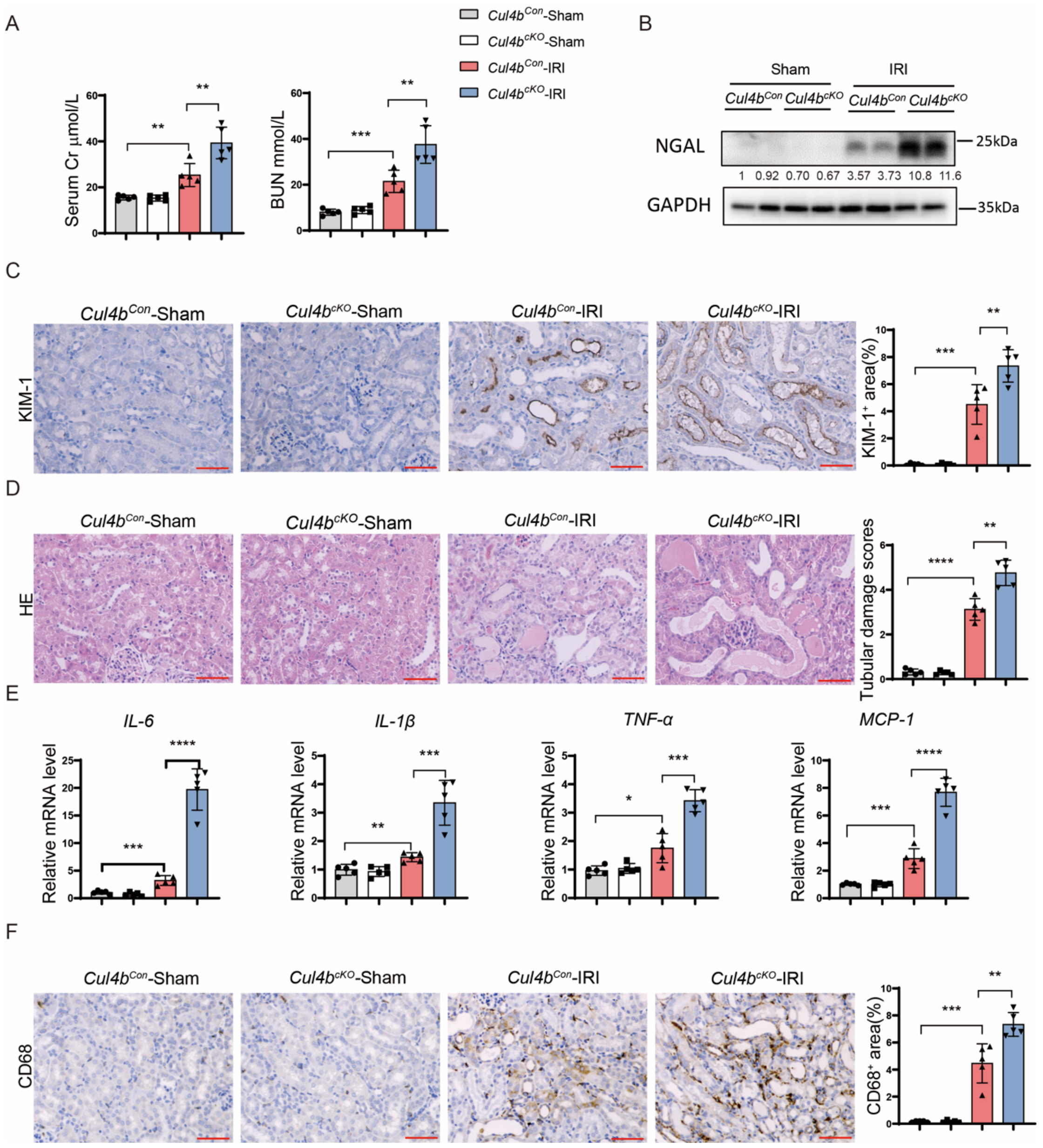

Supplementary figure S4. *Cul4b* deficiency exacerbates IR-induced AKI.

A) The creatinine (Cr) and BUN levels in the serum collected from the *Cul4b*^{con} or *Cul4b*^{cKO} mice at 72 hrs after IR or sham operation. N=5. B) Western blots of NGAL in the kidneys from the indicated mice. C) The representative images of immunohistochemistry staining of KIM-1 and quantification of the percentage of KIM-1^{+} area in the indicated kidneys. 5 fields were imaged for each mouse and 5 mice were included in each group. Scale bar, 60 μm. D) The representative images of H & E

staining and the tubular damage scores of the kidneys from the indicated mice. 10 fields were imaged for each mouse and 5 mice were included in each group. Scale bar, 60 μm. E) The mRNA levels of the indicated inflammatory factors in the kidneys. N=5. F) The representative images of immunohistochemistry staining of the macrophage marker CD68 and quantification of the percentage of $CD68^{+}$ area in kidneys. 5 fields were imaged for each mouse and 5 mice were included in each group. Scale bar, 60 μm. In all bar graphs, the grey bars represent $Cul4b^{Con}$ mice with sham operation ($Cul4b^{Con}$-Sham); the white bars represent $Cul4b^{cKO}$ mice with sham operation ($Cul4b^{cKO}$-Sham); the red bars represent $Cul4b^{Con}$ mice with IRI ($Cul4b^{Con}$-IRI); the blue bars represent $Cul4b^{cKO}$ mice with IRI ($Cul4b^{cKO}$-IRI). Data are presented as mean ± SD. *: $P< 0.05$; **: $P< 0.01$; ***: $P< 0.001$; ****: $P< 0.0001$.

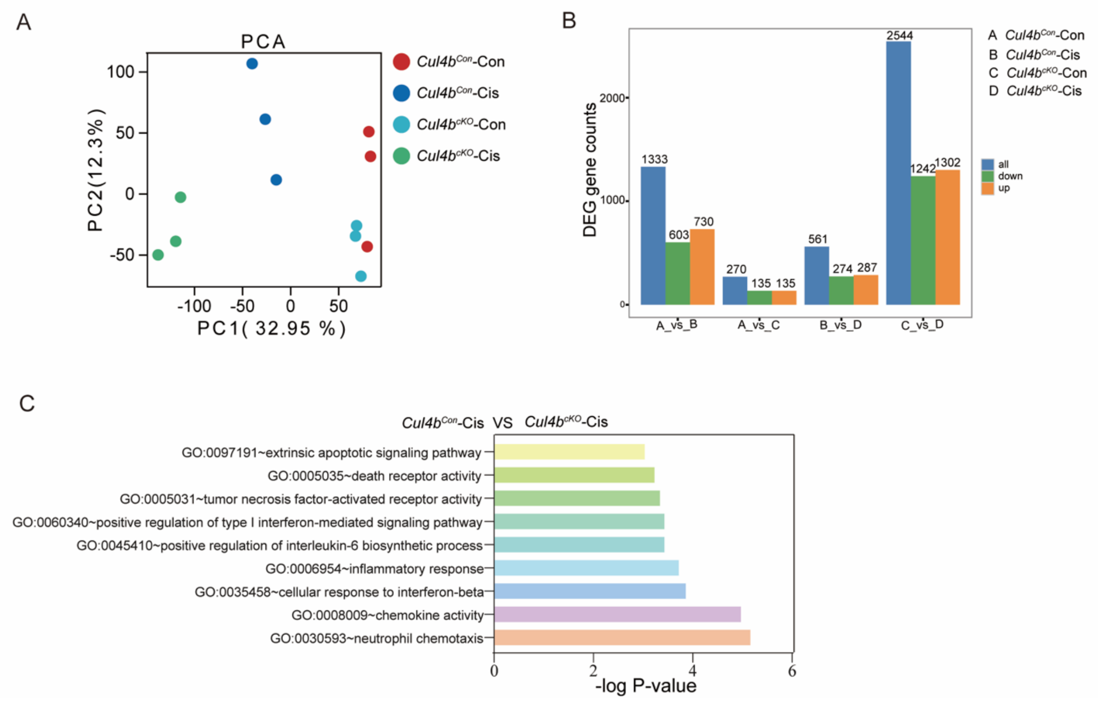

Supplementary figure S5. RNA sequencing of kidneys with cisplatin-induced AKI.

A) Principal component analysis of RNAseq data. B) Summary of differentially expressed genes. C) GO enrichment analysis showing the genes upregulated in cisplatin-treated *Cul4b*^{cKO} kidneys compared to cisplatin-treated *Cul4b*^{Con} kidneys are enriched in GO terms related to apoptosis and inflammation.

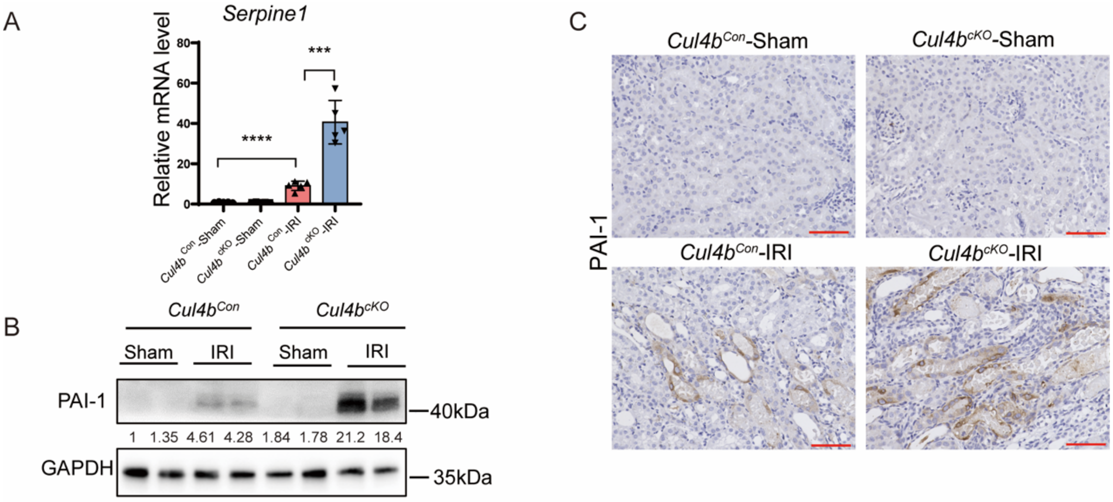

Supplementary figure S6. Loss of CUL4B induces upregulation of PAI-1 in kidneys after IRI.

A-B) The levels of *Serpine1* mRNA (A) and PAI-1 protein (B) in kidneys from the indicated groups. N=5 in (A). Data are presented as mean ± SD. *: *P*< 0.05; **: *P*< 0.01; ***: *P*< 0.001; ****: *P*< 0.0001. C) Immunohistochemistry staining of PAI-1 in kidneys from the indicated groups. Scale bar, 60 μm.

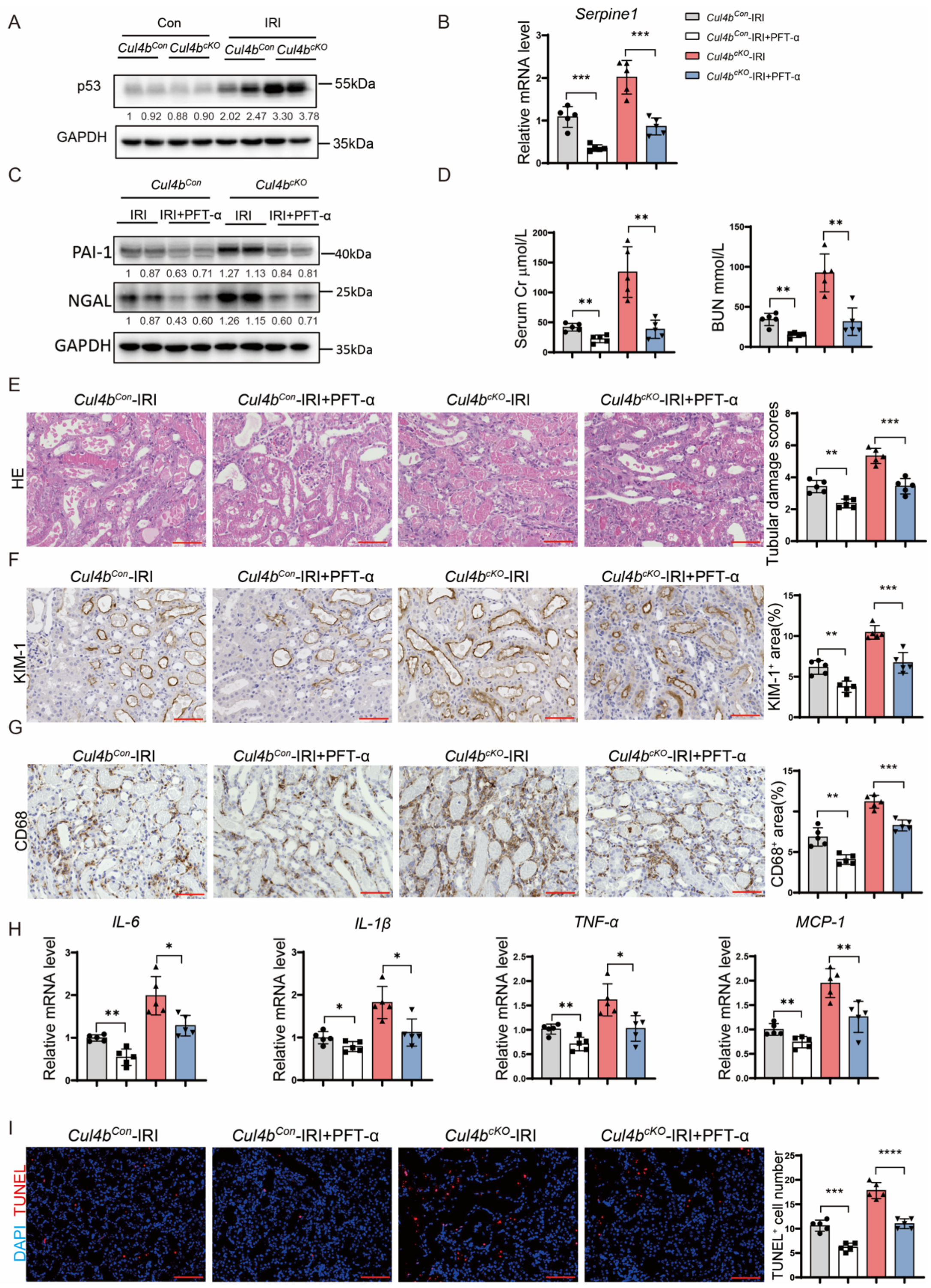

Supplementary figure S7. CUL4B suppresses PAI-1 expression after IRI by upregulating p53.

A) Western blots showing the level of p53 in the indicated kidneys. B) The level of *Serpine1* mRNA in kidneys from the *Cul4b*^{Con} or *Cul4b*^{cKO} mice with IRI and

pretreatment with PFT-α or vehicle control. N=5. C) Western blots showing the levels of PAI-1 and NGAL in the indicated kidneys. D) The creatinine (Cr) and BUN levels in the serum collected from the indicated mice. N=5. E) The representative images of H & E staining and the tubular damage scores of the kidneys from the indicated mice. 10 fields were imaged for each mouse and 5 mice were included in each group. Scale bar, 60 μm. F) The representative images of immunohistochemistry staining of KIM-1 in kidneys and the quantification of the percentage of KIM-1^+ area. 5 fields were imaged for each mouse and 5 mice were included in each group. Scale bar, 60 μm. G) The representative images of immunohistochemistry staining of the macrophage marker CD68 and quantification of the percentage of $CD68^+$ area in kidneys. 5 fields were imaged for each mouse and 5 mice were included in each group. Scale bar, 60 μm. H) The mRNA levels of the indicated pro-inflammatory factors in the kidneys. N=5. I) The representative images of TUNEL staining on kidneys and the quantification of the number of $TUNEL^+$ cells per field. 5 fields were imaged for each mouse and 5 mice were included in each group. Scale bar, 85 μm. In all bar graphs, the grey bars represent $Cul4b^{Con}$ mice with IRI and pretreatment with vehicle control ($Cul4b^{Con}$-IRI); the white bars represent $Cul4b^{Con}$ mice with IRI and pretreatment with PFT-α ($Cul4b^{Con}$-IRI+PFT-α); the red bars represent $Cul4b^{cKO}$ mice with IRI and pretreatment with vehicle control ($Cul4b^{cKO}$-IRI); the blue bars represent $Cul4b^{cKO}$ mice with IRI and pretreatment with PFT-α ($Cul4b^{Con}$-IRI+PFT-α). Data are presented as mean ± SD. *: $P< 0.05$; **: $P< 0.01$; ***: $P< 0.001$; ****: $P< 0.0001$.

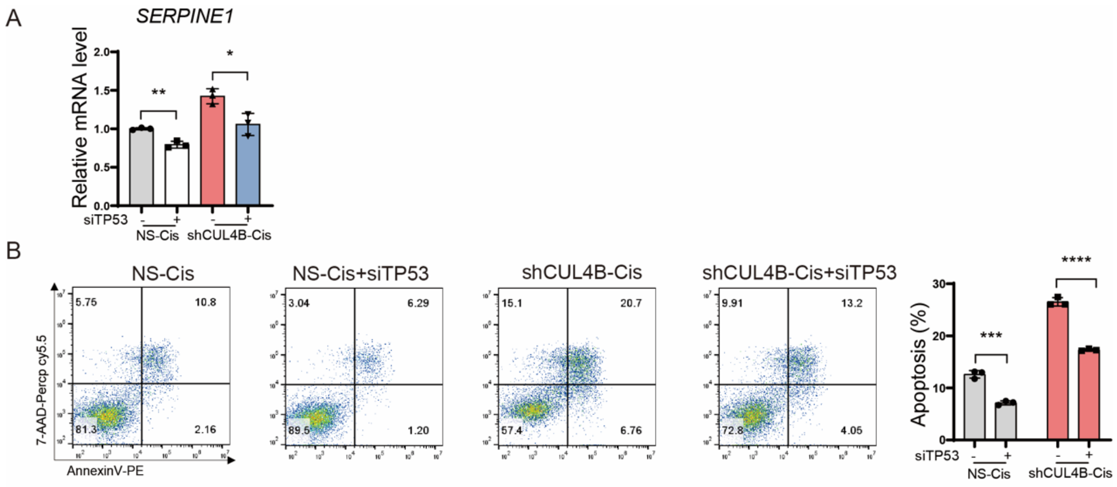

Supplementary figure S8. Knockdown of *TP53* suppresses cisplatin-induced *SERPINE1* upregulation and apoptosis in HK2 cells.

A) The level of *SERPINE1* mRNA in the cisplatin-treated HK2 cells expressing control shRNA (NS) or shRNA against *CUL4B* (shCUL4B) with or without expression of siRNA against *TP53* (siTP53). N=3. B) The representative images of Annexin V/7-AAD analysis of the indicated HK2 cells and the quantification of the percentage of Annexin V^{+} cells. N=3. Data are presented as mean ± SD. *: *P*< 0.05; **: *P*< 0.01; ***: *P*< 0.001; ****: *P*< 0.0001.

A

Peptide sequence	score	modification
AMAIY**K**QSQHMTEVVR	891	GG(K164)

B

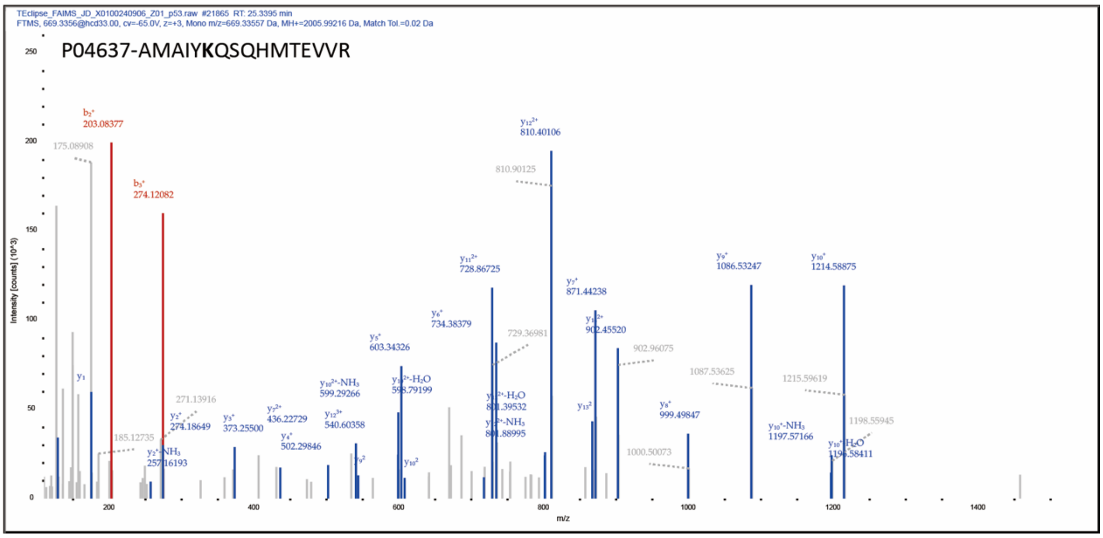

Supplementary figure S9. Mass spectrometry identifies K164 of p53 protein as the major ubiquitination target residue of CRL4B.

The di-glycine-modified K164 in p53 was detected, and the corresponding peptide was AMAIYKQSQHMTEVVR.

Supplementary table S1. List of antibodies

**Antigen name**	**Cat#**	**Supplier**
GAPDH	60004	Proteintech
CUL4B (for WB)	C9995	Sigma
CD68	97778S	Cell Signaling Technology
CUL4B (for IHC)	12916	Proteintech
NGAL	sc-515876	Santa Cruz Biotechnology
HAVCR1 (for IHC)	BA3537	BOSTER
Cleaved Caspase 3	9664S	Cell Signaling Technology
PAI-1	AB222754	Abcam
TIM-1/KIM-1/HAVCR (for WB)	MAB1817	R&D SYSTEMS
p53 (for WB)	sc-126	Santa Cruz Biotechnology
p53 (for IP)	sc-6243	Santa Cruz Biotechnology
HA Tag	66006	Proteintech
LTL, Fluorescein	FL-1321	Vector Laboratories
Calbindin D28K	sc-365360	Santa Cruz Biotechnology
Synaptopodin	AP33487SU-N	OriGene
Myc Tag	RA1005	Vazyme
IgG	2729S	Cell Signaling Technology

WB: Western blotting; IHC: immunohistochemistry; IP: immunoprecipitation

Supplementary table S2. List of primers and siRNA.

Primer name	Forward sequence	Reverse sequence
ACTB	CTACCTCATGAAGATCCTGACC	CACAGCTTCTCTTTGATGTCAC
IL6	TAGTCCTTCCTACCCCCAATTCC	TTGGTCCTTAGCCACTCCTTC
IL-1β	TGCCACCTTTTGACAGTGATG	TGATGTGCTGCTGCGAGATT
TNFα	GCCACCACGCTCTTCTGTCTAC	GAGAGGGAGGCCATTTGGGAAC
MCP1	CACTCACCTGCTGCTACTCATTC	GCTTCTTTGGGACACCTGCTG-
Serpine1	CGCCTGGTGCTGGTGAATGC	GACGGTGCTGCCATCAGACTTG
SERPINE1	GGTGCTGGTGAATGCCCTCTAC	CAGTGCTGCCGTCTGATTTGTG
Trp53	TGAACCGCCGACCTATCCTTAC	TCCCAGGGCAGGCACAAAC
TP53	CCTCCTCAGCATCTTATCCGAGTG	CCTCCTCAGCATCTTATCCGAGTG
K164R mutation	GGCCATCTACcgcCAGTCACAGCACATGACGG	ATGGCGCGGACGCGG
si*SERPINE1*	GAGCCAGAUUCAUCAUCAATT	UUGAUGAUGAAUCUGGCUCTT
si*TP53*	CCAUCUACAAGCAGUCACATT	UGUGACUGCUUGUAGAUGGTT
siRNA control	UUCUCCGAACGUGUCACGUTT	ACGUGACACGUUCGGAGAATT
