# Supplementary material for: CUL4B protects kidneys from acute injury by restraining p53/PAI-1 signaling
Source: Cell Death Dis. 2024 Dec 18;15(12):915. doi: 10.1038/s41419-024-07299-w (PMC11655551; doi:10.1038/s41419-024-07299-w)

Fig1-B

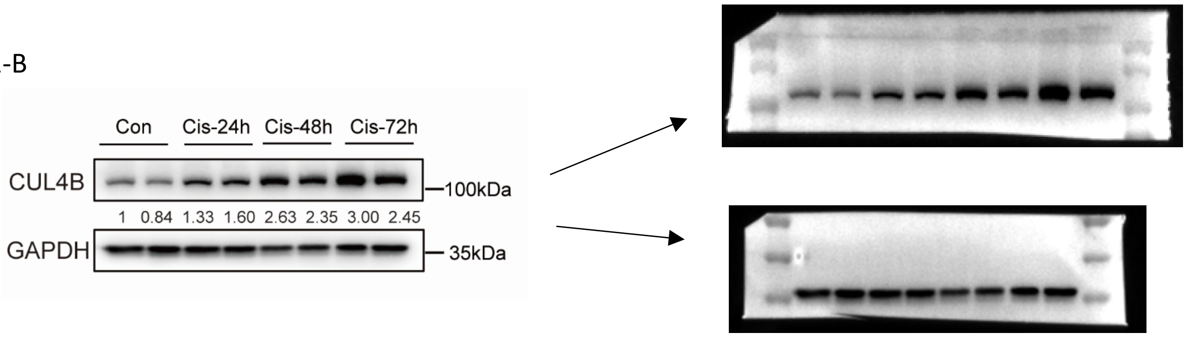

Fig1-E

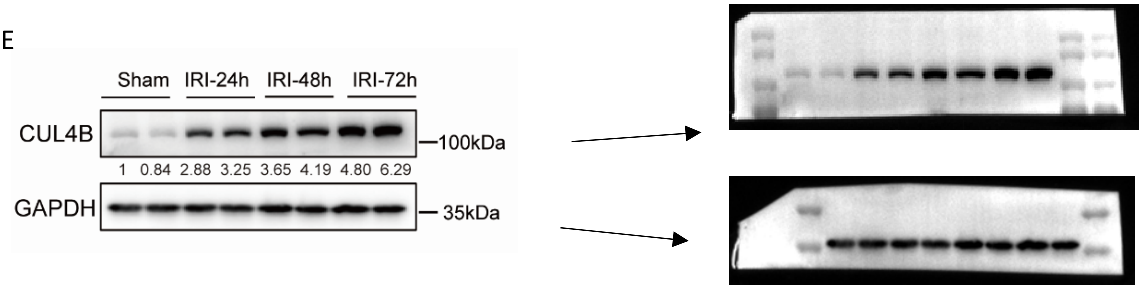

Fig2-B

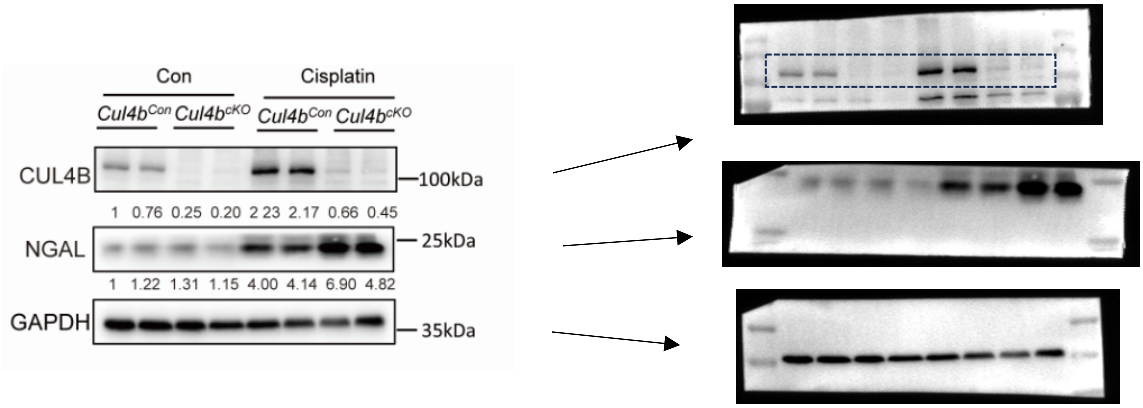

Fig3-C

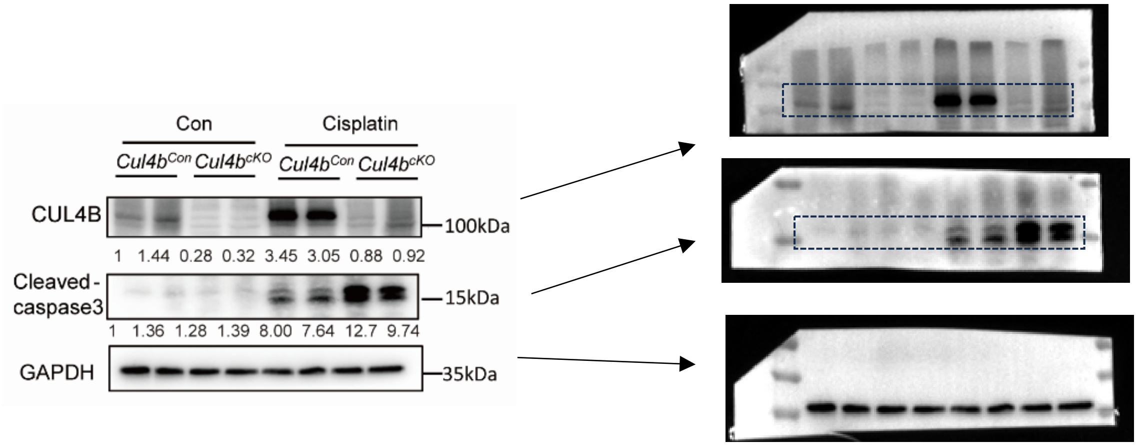

Fig3-D

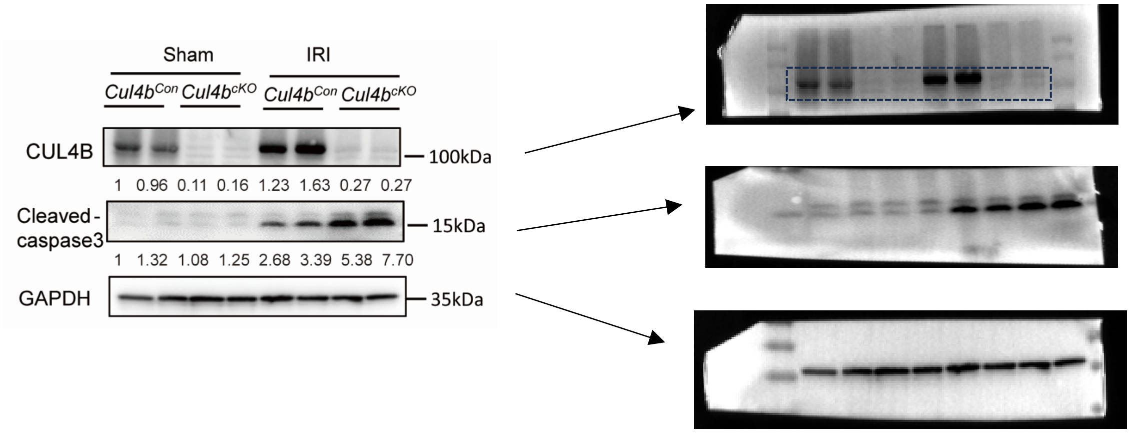

Fig3-E

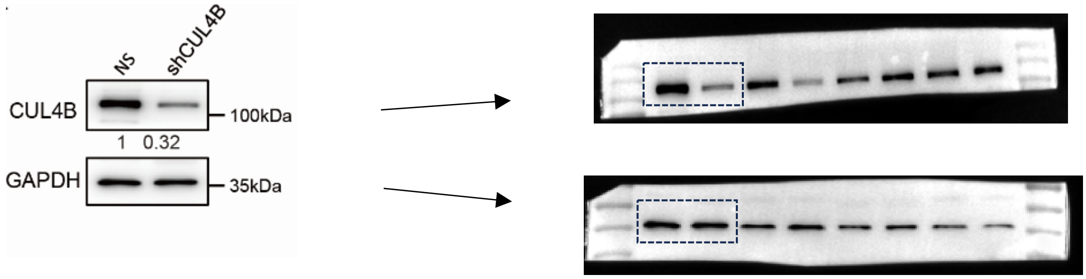

Fig4-B

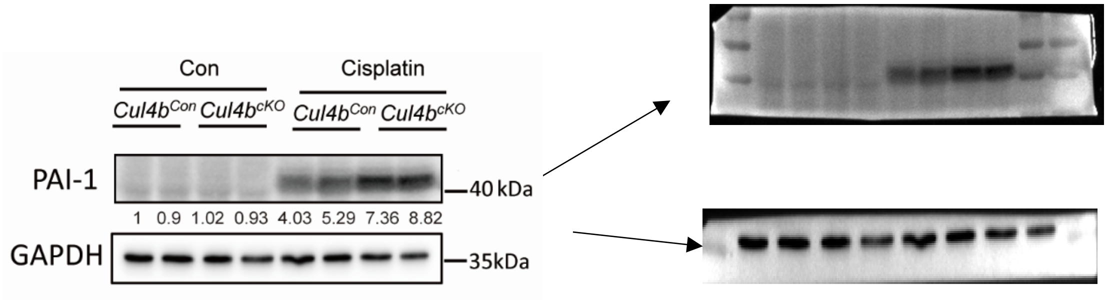

Fig4-E

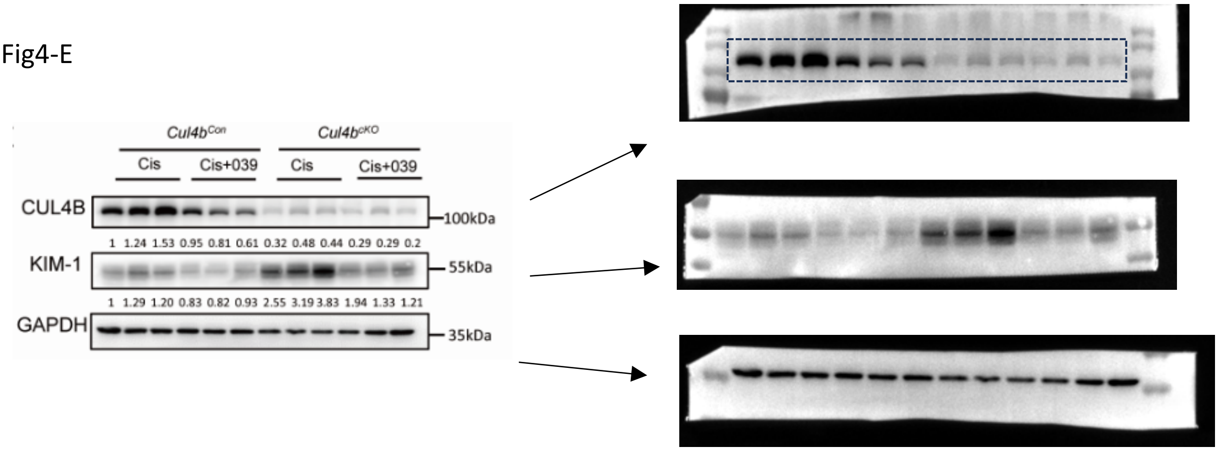

Fig5-A

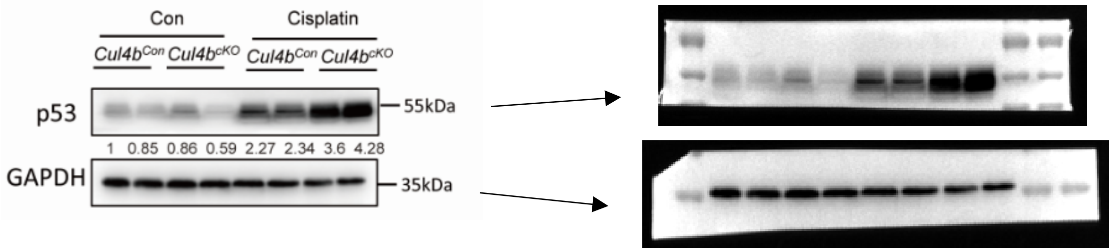

Fig5-C

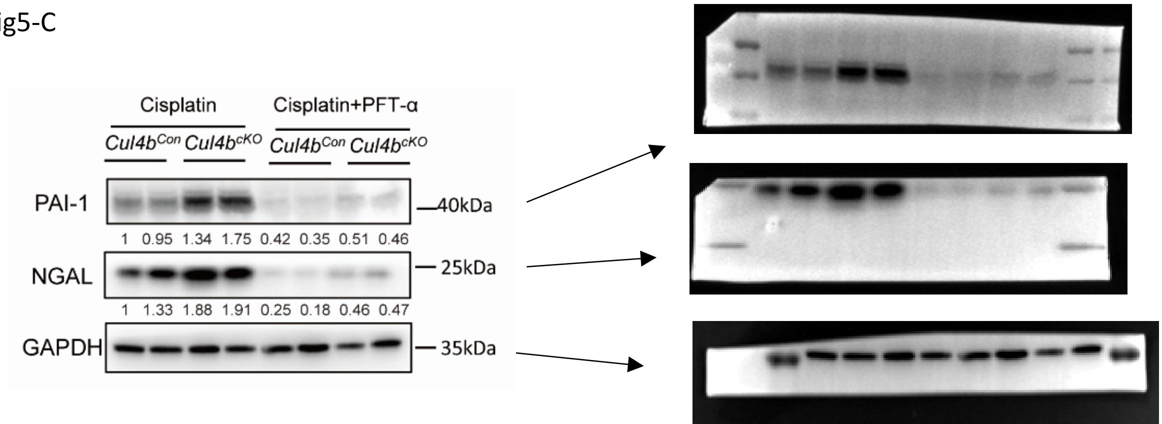

Fig6-C

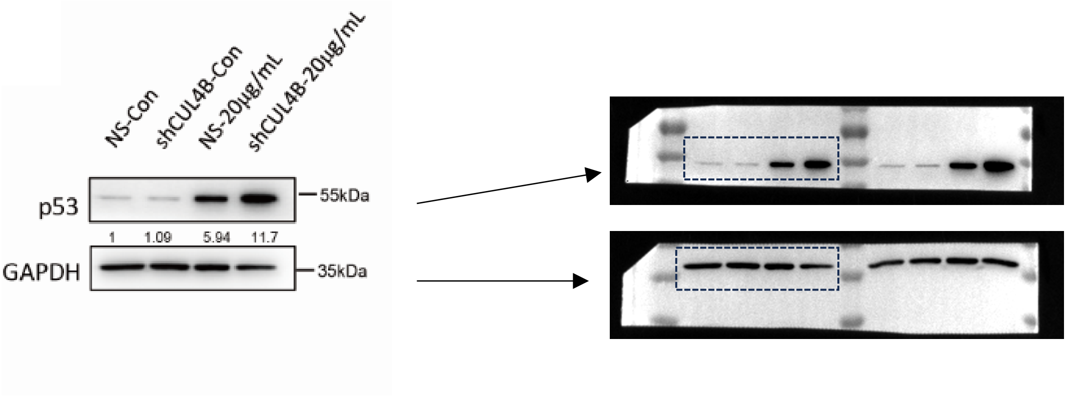

Fig6-D

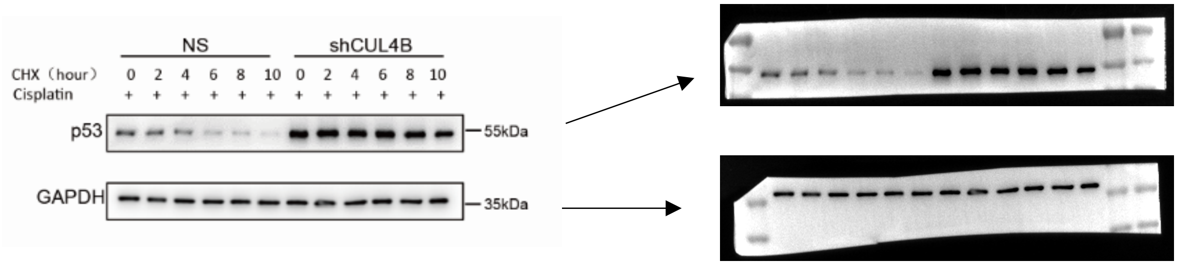

Fig6-E

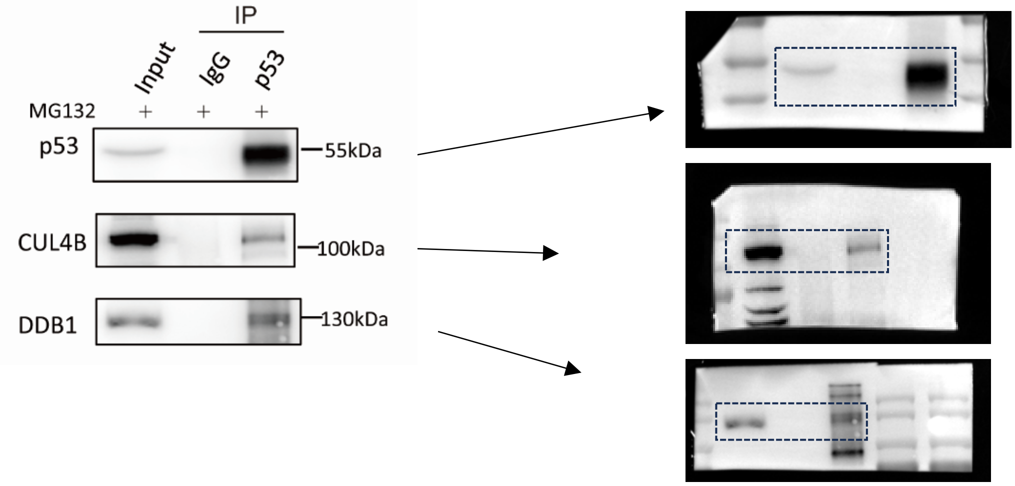

Fig6-F

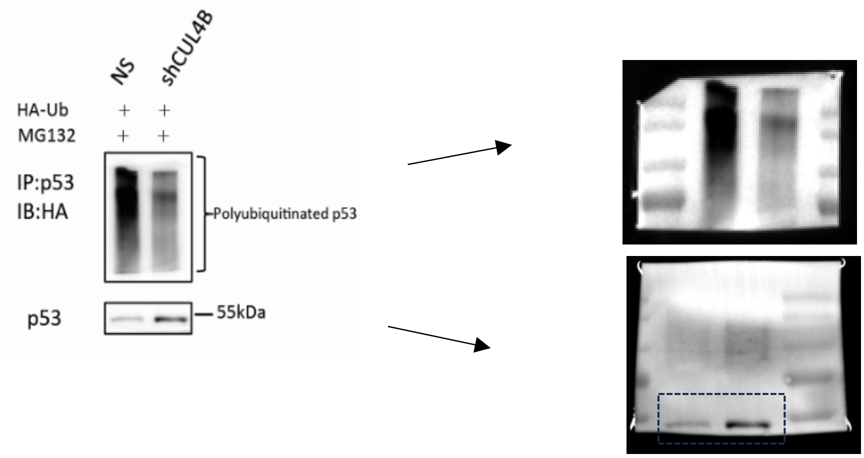

Fig6-G

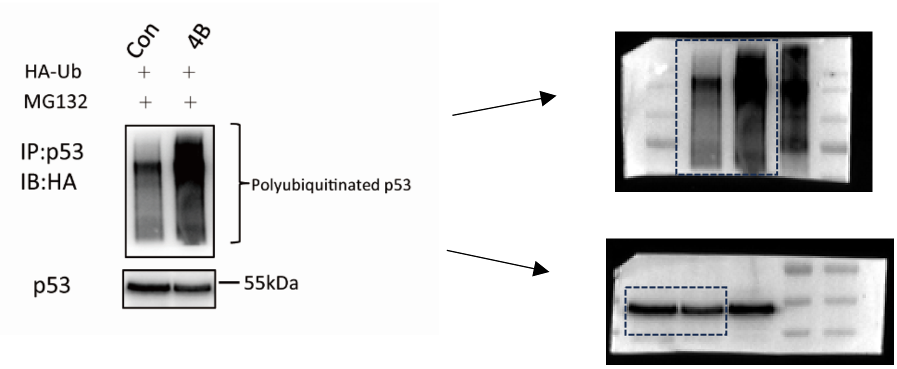

Fig6-H

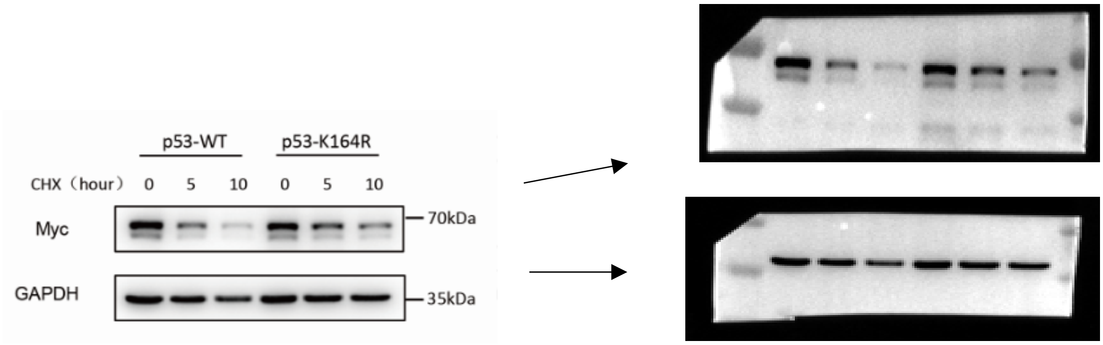

Fig6-I

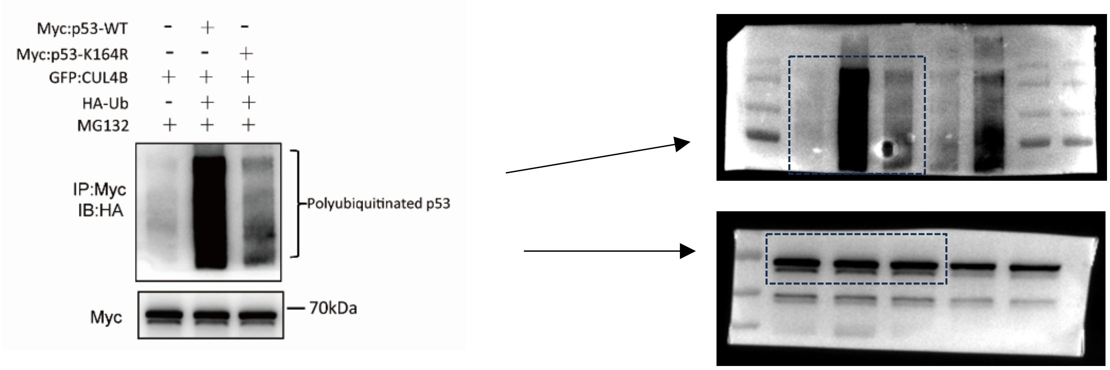

FigS2-C

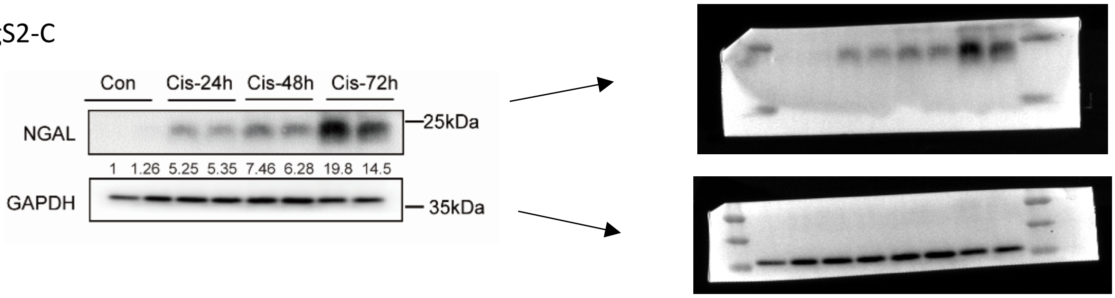

FigS2-D

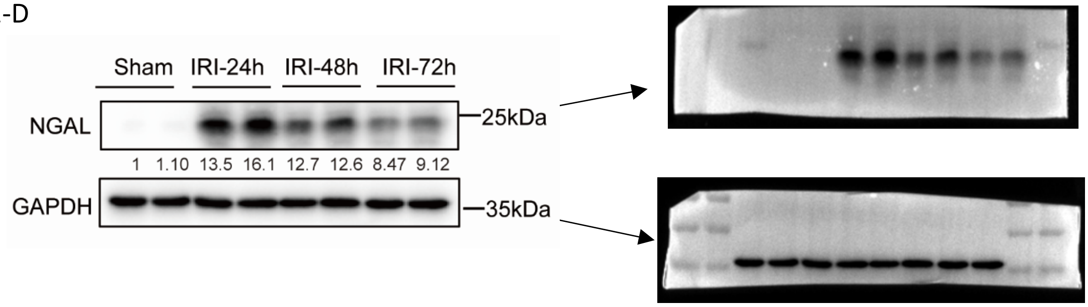

FigS4-B

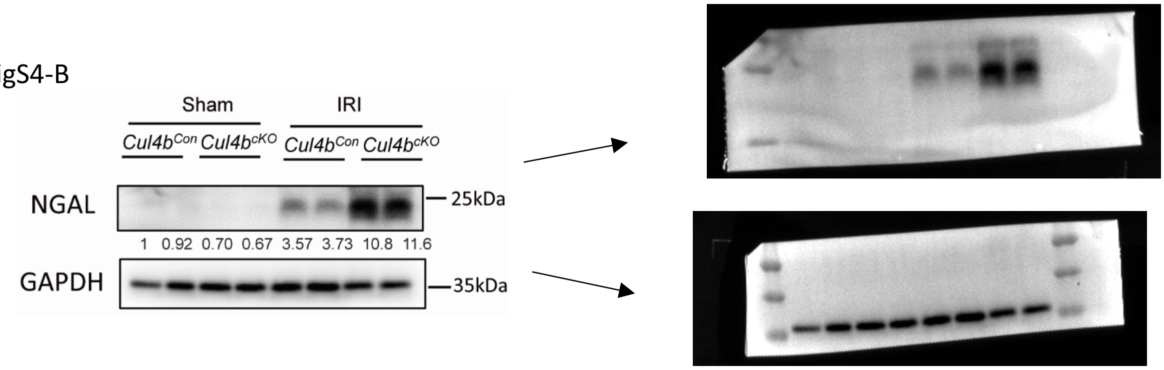

FigS6-B

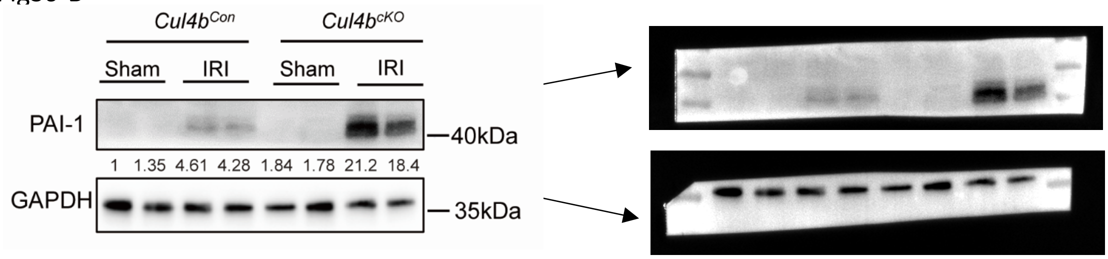

FigS7-A

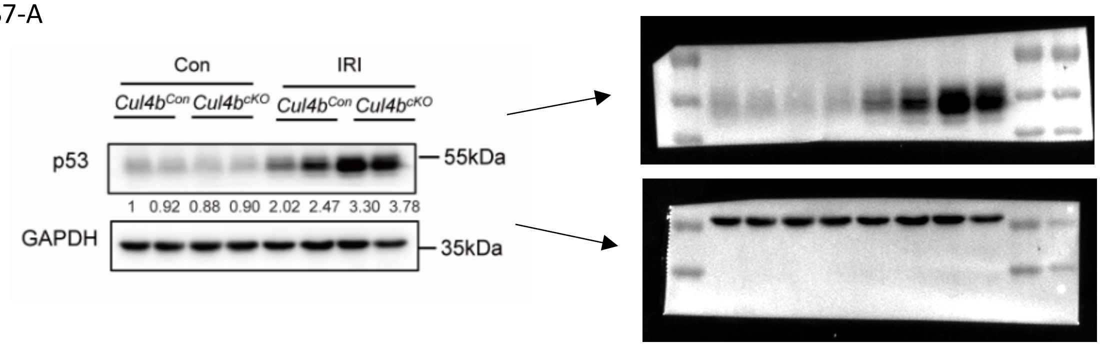

FigS7-C

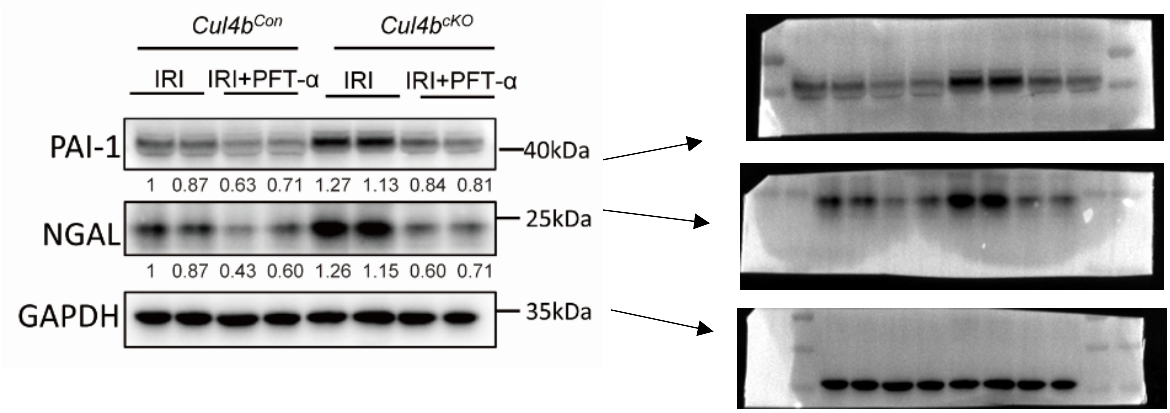

Supplement: Supplementary file 2 — original images of Western blots [file 41419_2024_7299_MOESM2_ESM.pdf]
